# FMT alleviates multidrug-resistant *Salmonella enterica*-induced diarrhea and is associated with loss of IncHI2A-associated resistance determinants in mice

**DOI:** 10.3389/fmicb.2026.1913730

**Published:** 2026-08-27

**Authors:** Panpan Xia, Huimin Wu, Wanzhao Chen, Daibing Wang, Shuqin Xu, Yujie Yang, Tianyuan Zeng, Lining Xia

**Affiliations:** 1 College of Veterinary Medicine, Xinjiang Agricultural University, Urumqi, China; 2 Xinjiang Key Laboratory of New Drug Research and Development for Herbivorous Animals (XJ-LNDRDHA), Urumqi, China

**Keywords:** antibiotic resistance genes, colonization resistance, fecal microbiota transplantation, gut microbiota, multidrug resistance, *Salmonella enterica*

## Abstract

**Introduction:**

Multidrug-resistant (MDR) *Salmonella enterica* (*S. enterica*) poses a serious threat to animal and public health because of increasingly limited treatment options. Fecal microbiota transplantation (FMT) is a potential microbiota-based intervention; however, its effects on MDR *Salmonella* infection and pathogen-associated antibiotic resistance gene (ARG) dynamics remain unclear.

**Methods:**

A murine diarrhea model was established using the clinical MDR *S. enterica* isolate P174, and infected mice were treated with FMT. Clinical symptoms, intestinal pathology, transcriptional inflammatory responses, gut microbiota composition, and ARG profiles of recovered *Salmonella* isolates were evaluated. Whole-genome sequencing was used to characterize resistance determinants, and the stability of ARGs and IncHI2A backbone markers was further assessed during 19 in vitro passages.

**Results:**

FMT reduced diarrhea, promoted body weight recovery, and alleviated intestinal tissue injury and inflammatory cell infiltration. Colonic expression of *Tnf*, *Il1b*, and *Il6* decreased, whereas *Il10* expression increased. FMT was also associated with partial recovery of gut microbial diversity, increased relative abundances of *Lactobacillus*, *Bifidobacterium*, and other commensal anaerobic taxa, and reduced *Salmonella* abundance. Whole-genome sequencing showed that *bla*_OXA-1_, *floR*, *oqxA*, and *oqxB* were co-localized on an IncHI2A-associated plasmid sequence. Loss of these resistance determinants increased over time in isolates recovered from FMT-treated mice, whereas no loss of the four ARGs or the IncHI2A backbone markers *repB* and *parB* was detected during 19 *in vitro* passages. Among isolates showing simultaneous loss of all four ARGs, nearly all also lacked detectable *repB* and *parB*, whereas isolates with partial ARG loss retained both markers. These patterns were consistent with both backbone-associated loss and resistance-region deletion or rearrangement. Most ARG-loss isolates showed reduced antimicrobial resistance.

**Discussion:**

FMT alleviated MDR *S. enterica*-induced intestinal disease and was associated with partial recovery of gut microbiota characteristics and increased instability and loss of IncHI2A-associated resistance determinants *in vivo*. These findings suggest a potential association between intestinal microbial ecological changes and altered maintenance patterns of resistance-associated genetic elements in MDR *S. enterica*.

## 1 Introduction

The rapid emergence and global dissemination of antimicrobial resistance (AMR) have become major threats to public health, food safety, and animal production systems. Among foodborne pathogens, multidrug-resistant (MDR) *Salmonella enterica* (*S. enterica*) is of particular concern due to its zoonotic transmission potential and its ability to acquire and disseminate antibiotic resistance genes (ARGs) via plasmids, integrons, and other mobile genetic elements (Michael and Schwarz, 2016; Partridge et al., 2018). Increasing resistance to critically important antimicrobials, including fluoroquinolones and third-generation cephalosporins, has significantly limited treatment options for both human and veterinary infections (Sati et al., 2025). These challenges highlight the urgent need for alternative, non-antibiotic strategies to control MDR *Salmonella* infections.

The intestinal microbiota plays a central role in maintaining host health and protecting against pathogen colonization through a process known as colonization resistance. Commensal microbial communities inhibit pathogen expansion via multiple mechanisms, including nutrient competition, production of antimicrobial metabolites, and modulation of host immune responses (Buffie and Pamer, 2013; Ducarmon et al., 2019). However, antibiotic exposure and MDR pathogen infection can disrupt this ecological balance, leading to dysbiosis, reduced colonization resistance, and increased persistence of resistant bacteria. Importantly, the gut microbiota also serves as a major reservoir of ARGs, contributing to the maintenance and dissemination of antimicrobial resistance within the host (Sommer et al., 2009). In addition to influencing pathogen abundance, changes in the intestinal ecological environment may affect the selective pressures acting on mobile resistance elements carried by colonizing bacteria. Restoration of the gut microbiota may therefore represent an ecological approach not only to suppress MDR pathogens but also to influence the stability of their associated resistance determinants.

Fecal microbiota transplantation (FMT), which involves the transfer of complex microbial communities from healthy donors to recipients, has emerged as an effective strategy for restoring gut microbial homeostasis. While FMT is well established in the treatment of recurrent *Clostridioides difficile* (*C. difficile*) infection, accumulating evidence suggests that it may also contribute to the decolonization of multidrug-resistant organisms (MDROs) and the reduction of ARG abundance within the gut resistome. For instance, Leung et al. reported significant depletion of clinically relevant ARGs following FMT in patients with recurrent *C. difficile* infection (Leung et al., 2018). Similarly, recent studies have demonstrated that microbiota-based interventions can reshape the gut resistome and influence resistance gene dynamics (Rashidi et al., 2024). In veterinary medicine, FMT has shown potential in improving growth performance, alleviating diarrhea, and restoring gut microbiota in livestock and companion animals (Reveles et al., 2025). Nevertheless, reductions in the overall gut resistome do not necessarily indicate that resistance determinants have been lost from an established MDR pathogen. Most previous studies have focused on community-level ARG abundance, whereas whether FMT influences the stability of pathogen-associated resistance determinants remains unknown. In particular, it is unclear whether restoration of the gut microbiota affects the *in vivo* stability of IncHI2A-associated resistance determinants in MDR *S. enterica*.

In this study, we established a murine diarrhea model using the clinical MDR *S. enterica* isolate P174 to evaluate the effects of FMT on disease severity, intestinal pathology, inflammatory gene expression, and gut microbiota restoration. We further investigated the dynamics of IncHI2A-associated resistance determinants in *Salmonella* isolates recovered from mice and compared these findings with those from *in vitro* serial passage. We hypothesized that FMT-mediated microbiota restoration would alleviate infection and promote instability of IncHI2A-associated resistance determinants. This study therefore extends previous community-level resistome analyses by examining resistance determinant dynamics within MDR *S. enterica in vivo*.

## 2 Materials and methods

### 2.1 Genomic extraction and whole-genome sequencing

Whole-genome sequencing (WGS) of the MDR *S. enterica* strain P174 was performed to characterize its genomic features and resistance determinants. Genomic DNA was extracted using the FastPure® Bacterial DNA Isolation Mini Kit according to the manufacturer’s instructions. DNA integrity was verified by 1% agarose gel electrophoresis, and DNA concentration and purity were measured using a NanoDrop spectrophotometer. Species identification and preliminary detection of antibiotic resistance genes were conducted by PCR amplification prior to sequencing.

Qualified genomic DNA samples were subjected to short-read sequencing (2 × 150 bp paired-end) using the Illumina HiSeq 2500 platform, and long-read sequencing was performed using the BGI CycloneSEQ-WT02 platform. Short-read assembly was initially performed using SPAdes v3.14.0, while long-read assembly was conducted using Flye v2.4.2. Hybrid assembly integrating both short-read and long-read data was subsequently performed using Unicycler v0.4.8 to improve genome continuity and resolve complex genomic structures, including plasmids. Genome annotation was performed using Prokka v1.15.6. Assembly quality was evaluated using QUAST v5.3.0 based on contig number, total assembly length, N50, L50, and GC content. Genome completeness and contamination were assessed using CheckM2 v1.1.0. Plasmid replicon types and mobility-related characteristics were analyzed using MOB-suite (MOB-typer v3.1.0). ARGs were identified using the Resistance Gene Identifier (RGI) v6.0.5 pipeline against the Comprehensive Antibiotic Resistance Database (CARD) v4.0.1. Candidate ARGs were retained according to CARD-defined criteria based on model-specific bit-score thresholds, sequence identity, and reference sequence coverage. The circular plasmid map was generated using the Proksee web server (Grant et al., 2023).

### 2.2 Animals

Female BALB/c mice, aged 6–8 weeks, were purchased from Xinjiang Medical University. All mice were housed individually under standard laboratory conditions with a 12 h light/dark cycle and allowed to acclimatize for 1 week before experimentation. Feed, bedding, and water were sterilized prior to use, and cages were regularly disinfected using ultraviolet (UV) light. All experimental procedures were approved by the Laboratory Animal Welfare Ethics Committee of Xinjiang Agricultural University (Approval No. 2020018).

### 2.3 Animal model, experimental design, and treatment

(i) A total of 65 mice were used, including 60 recipient mice and 5 donor mice. Donor mice were obtained from the same animal batch and matched with recipient mice in terms of strain, sex, and age. Recipient mice were randomly assigned into four groups (*n* = 15 per group): FMT treatment group (FMT), antibiotic treatment group (Abx), untreated infected group (Unt), and healthy control group (Ctrl). Donor mice were used exclusively for fecal microbiota preparation.
(ii) Infection model. To facilitate bacterial colonization, mice in the FMT, Abx, and Unt groups received an antibiotic cocktail (ampicillin, metronidazole, and neomycin; 1 g/L each) in drinking water for 7 consecutive days prior to infection. The Ctrl group received untreated drinking water. Following antibiotic pretreatment, mice in the FMT, Abx, and Unt groups were orally gavaged with 0.2 mL of 3% sodium bicarbonate to neutralize gastric acidity. After 15 min, mice were challenged with MDR *S. enterica* P174 at a dose of approximately 1 × 10^8^ CFU per mouse. The Ctrl group received sterile MH broth at an equivalent volume.
(iii) FMT preparation and administration. Donor mice were healthy, untreated mice maintained under the same specific pathogen-free conditions as recipient mice. Donor mice did not receive antibiotic treatment throughout the experiment and were used exclusively for fecal microbiota preparation. FMT treatment was initiated after infection and administered once daily by oral gavage for 5 consecutive days. Prior to each administration, 0.2 mL of 3% sodium bicarbonate was given to reduce gastric acidity. Fresh feces from donor mice were collected within 3 h, pooled, weighed, and suspended in sterile physiological saline at a concentration of 250 mg/mL (wet weight). The suspension was homogenized and used immediately without filtration or storage to preserve microbial viability. The entire preparation process was completed within 2 h. No anaerobic conditions were applied during preparation. The fecal suspension was administered at 0.1 mL per 10 g body weight. Before transplantation, donor fecal samples were screened by PCR and culture-based methods and confirmed negative for *Salmonella* and the target antibiotic resistance genes (*bla*_OXA-1_, *floR*, *oqxA*, and *oqxB*). Donor fecal samples were pooled prior to FMT administration. The donor microbiota composition was not independently characterized by 16S rRNA gene sequencing.
(iv) Antibiotic treatment regimen. Mice in the Abx group received intramuscular amikacin twice daily (10:00 and 22:00) for 5 consecutive days. Amikacin was administered at a dose of 0.15 mg per mouse per injection. This antibiotic was selected based on the susceptibility profile of strain P174 (MIC = 4 μg/mL, according to CLSI criteria). Mice in the Unt and Ctrl groups received no treatment during this period.

After the treatment phase, all mice were maintained under standard conditions for a 7-day recovery period without further intervention. Fecal samples were collected daily throughout the experiment. Mice were euthanized at designated time points by cervical dislocation performed by trained personnel. All procedures were conducted in accordance with the approved protocols of the Laboratory Animal Welfare Ethics Committee of Xinjiang Agricultural University (Approval No. 2020018), and samples were collected for microbiota, histopathological, immunological, and molecular analyses.

### 2.4 Histopathological examination

Standardized sampling of tissues was performed to collect representative anatomical regions from the intestinal segments (duodenum, ileum, cecum, colon). Fresh tissues were fixed in 4% paraformaldehyde for 48 h, then processed through dehydration, paraffin embedding, and microtome sectioning. Tissue sections were stained with hematoxylin and eosin (H&E) following standard protocols. Histopathological evaluation was subsequently performed under light microscopy.

Histopathological changes were evaluated using a semiquantitative scoring system. Duodenal injury was assessed based on villus integrity, crypt architecture, epithelial integrity, inflammatory cell infiltration, and mucosal edema/congestion. Colonic injury was evaluated based on mucosal architecture integrity, crypt architecture, epithelial integrity, inflammatory cell infiltration, and mucosal edema/congestion. Each parameter was scored from 0 to 4 according to predefined criteria, with higher scores indicating more severe tissue injury. The detailed scoring criteria are provided in Supplementary Table S1. Histological scoring was independently performed by an investigator blinded to experimental group allocation. Three biological samples were evaluated per group (*n* = 3/group).

### 2.5 Analysis of inflammation-associated gene expression in murine colonic tissues

Target gene sequences were retrieved from the NCBI Nucleotide database.^1^ Primers were designed using Primer 5.0, and their specificity was verified using DNAMAN 9.0. Detailed primer sequences are listed in Supplementary Table S2.

Total RNA was extracted from murine colonic tissues using TRIzol reagent (TransGen Biotech, Beijing, China), and reverse transcription was performed using the EasyScript One-Step gDNA Removal and cDNA Synthesis Kit (TransGen Biotech). Reverse-transcription quantitative PCR (RT-qPCR) was ugsed to assess the relative mRNA expression of inflammation-associated genes, including *Il6* (encoding IL-6), *Il10* (encoding IL-10), *Il1b* (encoding IL-1β), and *Tnf* (encoding TNF-α), with *Actb* (β-actin) used as the reference gene. All reactions were performed in technical triplicate. Relative mRNA expression levels were calculated using the 2^-∆∆Ct^ method, with *β*-actin serving as the internal control.

### 2.6 Intestinal microbiota analysis

Luminal contents from the small intestine and colon were collected separately from each mouse immediately after euthanasia. Samples were placed into sterile tubes and stored for 16S rRNA gene sequencing. Each sample represented one mouse, and no samples were pooled. Three mice from each experimental group were randomly selected as independent biological replicates for microbiota profiling.

A total of 78 samples were sequenced, including samples from the small intestine and colon. Sequencing was performed by Shanghai Meiji Biomedical Technology Co., Ltd. (Shanghai, China) using an Illumina paired-end sequencing platform. After regrouping based on treatment type and sampling time, mice were categorized into eight experimental groups: Abx_Pre (antibiotic pretreatment), SE (*S. enterica*-infected), FMT (FMT-treated infected), Abx_Tx (antibiotic-treated infected), Unt (untreated infected), FMT_Rec (FMT-treated recovery), Abx_Rec (antibiotic -treated recovery), and Unt_Rec (untreated infected recovery). Healthy control samples were further divided into five time-matched subgroups: Ctrl_D0 (day 0), Ctrl_D1 (day 7), Ctrl_D2 (day 14), Ctrl_D3 (day 19), and Ctrl_D4 (day 26).

Bacterial genomic DNA was extracted using the FastPure® Bacterial DNA Isolation Mini Kit (Vazyme Biotech, Nanjing, China). The V3–V4 regions of the bacterial 16S rRNA gene were amplified using primers 338F and 806R. PCR amplification was performed in triplicate using TransStart FastPfu DNA Polymerase on an ABI GeneAmp® 9700 thermal cycler. Amplicons were purified, quantified, and pooled at equal concentrations for library construction using the TruSeq™ DNA Sample Prep Kit.

Raw paired-end reads were quality-filtered, merged, and demultiplexed. OTUs were clustered at 97% sequence similarity using USEARCH v11.0, and chimeric sequences were identified and removed using the UCHIME algorithm implemented in USEARCH v11.0. Chimeric sequences were removed using UCHIME. Representative sequences were used for taxonomic annotation. Taxonomic classification was performed using the RDP Classifier v2.13 against the SILVA database Release 138, with a confidence threshold of 0.70. Alpha diversity indices, including Chao1, Shannon, Simpson, and Pielou, were calculated using Mothur v1.30.2. Beta diversity was assessed by principal coordinate analysis based on Bray–Curtis distances using QIIME v1.9.1. Differences in microbial community structure were tested using PERMANOVA with 9,999 permutations. Differentially abundant taxa were identified using LEfSe v1.1.2 with an LDA score threshold of 2.0 and *p* < 0.05. PICRUSt2 v2.6.2 was used on the Majorbio Cloud Platform to predict KEGG functional profiles from 16S rRNA gene sequencing data. The exported pathway abundance tables were analyzed and visualized using R version 4.4.2 within the RStudio 4.6.0 environment.

### 2.7 Detection of antibiotic resistance gene loss in Salmonella isolates

Fecal samples from the FMT, Abx, and Unt groups were collected at three key time points: day 7 post-infection, day 5 post-intervention, and the final sampling day. A subset of samples from each group and time point was selected for bacterial isolation and analysis. Samples were homogenized in sterile MH broth and enriched in Rappaport–Vassiliadis (RV) medium supplemented with vancomycin (20 μg/mL) and linezolid (20 μg/mL) at 37 °C with shaking (180 rpm) for 48 h. Enriched cultures were subjected to serial tenfold dilutions and plated on *Salmonella*–Shigella (SS) agar, followed by incubation at 37 °C for 18 h. From each plate, 48 individual colonies were randomly selected and inoculated into 96-well plates containing MH broth. Isolates were then replica-plated onto SS agar without antibiotics and SS agar supplemented with ciprofloxacin (1 μg/mL), enrofloxacin (1 μg/mL), florfenicol (32 μg/mL), or ampicillin (32 μg/mL). Colonies that grew on antibiotic-free plates but failed to grow on antibiotic-containing plates were considered to have lost phenotypic resistance. These isolates were further cultured overnight, and genomic DNA was extracted for PCR confirmation. PCR amplification was performed to detect the *Salmonella*-specific gene *invA*, resistance-associated genes (*oqxA*, *oqxB*, *floR*, and *bla*_OXA-1_), and IncHI2A plasmid backbone-associated genes (*repB* and *parB*) (Supplementary Table S2). Loss of antibiotic resistance genes was defined as the absence of corresponding ARGs in isolates exhibiting reduced phenotypic resistance. The relationship between ARG loss and IncHI2A-associated genetic element stability was evaluated by analyzing the presence of plasmid backbone markers. Absence of both *repB* and *parB* was interpreted as loss of detectable IncHI2A backbone-associated regions, whereas retention of these markers indicated persistence of the plasmid backbone despite ARG loss.

### 2.8 *In vitro* serial passage and stability assessment of IncHI2A-associated resistance determinants

To determine whether ARG loss and IncHI2A-associated genetic element instability occurred spontaneously under laboratory conditions, the original challenge strain *Salmonella enterica* P174 was subjected to serial passage *in vitro*. Briefly, the strain was streaked onto SS agar plates and incubated at 37 °C for 18 h. A single colony was selected and inoculated into MH broth, followed by incubation at 37 °C with shaking at 180 rpm for 24 h to obtain the first-generation culture. Subsequently, 10 μL of the overnight culture was transferred daily into fresh MH broth under the same incubation conditions. Serial passaging was continued for 19 generations to match the duration of the *in vivo* infection experiment. Bacterial cultures from the 7th, 12th, and 19th generations were collected, mixed with sterile glycerol, and stored at −80 °C for subsequent analysis of antibiotic resistance gene stability. At generations 7, 12, and 19, 48 individual colonies were randomly selected from each culture and screened by PCR for the presence of *bla*_OXA-1_, *floR*, *oqxA*, *oqxB*, *repB*, and *parB*.

### 2.9 Statistical analysis

Data are presented as mean ± standard deviation (SD) or median (interquartile range) where appropriate. Statistical analyses were performed using SPSS v27.0, GraphPad Prism v9.3.0, and R software (v4.4.2). Data distribution and variance homogeneity were assessed using the Shapiro–Wilk test and Levene’s test, respectively. Parametric data were analyzed using independent-samples *t*-test or one-way ANOVA followed by Tukey’s HSD *post hoc* test. Non-parametric or ordinal data were analyzed using the Mann–Whitney U test or Kruskal–Wallis test followed by Dunn’s multiple comparison test with Holm correction. Histopathological scores of the duodenum and colon were analyzed separately. Microbial community differences were evaluated using PERMANOVA (Adonis) based on Bray–Curtis distance matrices with 9,999 permutations. Alpha-diversity indices were compared using appropriate statistical tests, and effect sizes were calculated where applicable. Functional profiles were predicted using PICRUSt2 on the Majorbio Cloud Platform, and predicted pathway abundances were further analyzed in R using pairwise Wilcoxon rank-sum tests with multiple-testing correction. All statistical tests were two-sided, and adjusted *p* values <0.05 were considered statistically significant. Non-significant differences were interpreted as exploratory trends.

## 3 Results

### 3.1 Genomic characterization of *Salmonella enterica* P174

Whole-genome sequencing identified strain P174 as *S. enterica*, belonging to sequence type ST34. Hybrid assembly generated a high-quality genome assembly consisting of five contigs, with a total length of 5,085,986 bp. Genome quality assessment showed 99.99% completeness and 0.19% contamination (Supplementary Table S3), supporting the reliability of downstream genomic characterization. As shown in Supplementary Table S4, the genome harbored 17 antibiotic resistance genes spanning eight antimicrobial classes, indicating a multidrug-resistant profile. Virulence gene analysis (Supplementary Table S5) identified 107 virulence-associated genes, including genes associated with *Salmonella* pathogenicity islands (*SPI-1*, *SPI-2*, and *SPI-3*).

**Figure 1:**
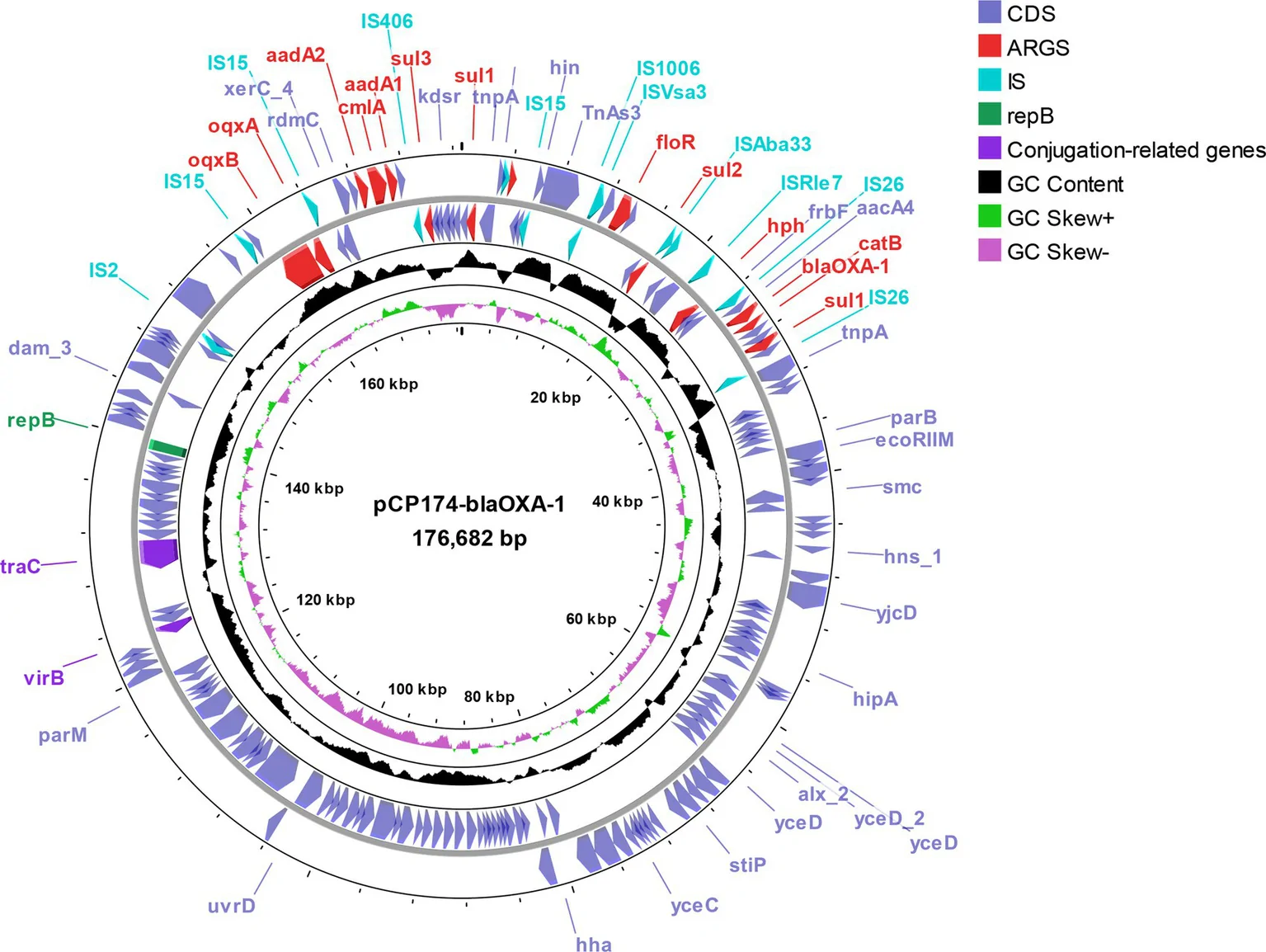
Circular map of the IncHI2A-type plasmid pCP174-*bla*_OXA-1_ (176,682 bp) from MDR *S. enterica* P174. Outer rings: Coding sequences (blue), antibiotic resistance genes (red; *bla*_OXA-1_, *floR*, *oqxA*, *oqxB*), insertion sequences (cyan; IS*15*, IS*26*, etc.), conjugation-associated genes (purple; *traC*, *virB*). Inner rings: GC content (black) and GC skew (green/purple).

Plasmid analysis revealed the presence of three replicon types, namely IncHI2A_1, IncHI2_1, and pKPC-CAV1321_1 (Supplementary Table S6). Further characterization of the IncHI2A plasmid identified a circular element of 176,682 bp, designated pCP174-*bla*_OXA-1_ (Figure 1). This plasmid contained a multidrug resistance region comprising four target resistance genes (*bla*_OXA-1_, *floR*, *oqxA*, and *oqxB*), together with additional resistance determinants, including *sul1*, *sul2*, *sul3*, *aadA1*, *aadA2*, *cmlA*, *catB*, and *aacA4*. These resistance genes were interspersed with multiple insertion sequences (IS*15*, IS*406*, IS*26*, IS*Aba33*, and IS*Rle7*), forming a region enriched in mobile genetic elements. In addition, the plasmid also carried several genes associated with conjugative transfer, including *traC* and *virB*-related genes, together with plasmid maintenance genes such as *parB*–*parM* and *repB*. A total of 13 mobile genetic elements were also identified across the genome (Supplementary Table S7). Overall, resistance genes in strain P174 were organized in clustered regions associated with mobile genetic elements, and the corresponding plasmid structures contained genes involved in transfer and stable maintenance.

### 3.2 Verification of gastrointestinal colonization and clinical outcomes

*Salmonella* was undetectable in all recipient groups before inoculation and remained absent in the healthy Ctrl group throughout the study. Following challenge with strain P174, the fecal recovery rate of *Salmonella* in infected mice reached 99.8% (Supplementary Table S8), indicating stable colonization. Whole-genome sequencing of one randomly selected fecal isolate identified it as *S. enterica* ST34, with a resistance gene profile consistent with the original challenge strain.

**Figure 2:**
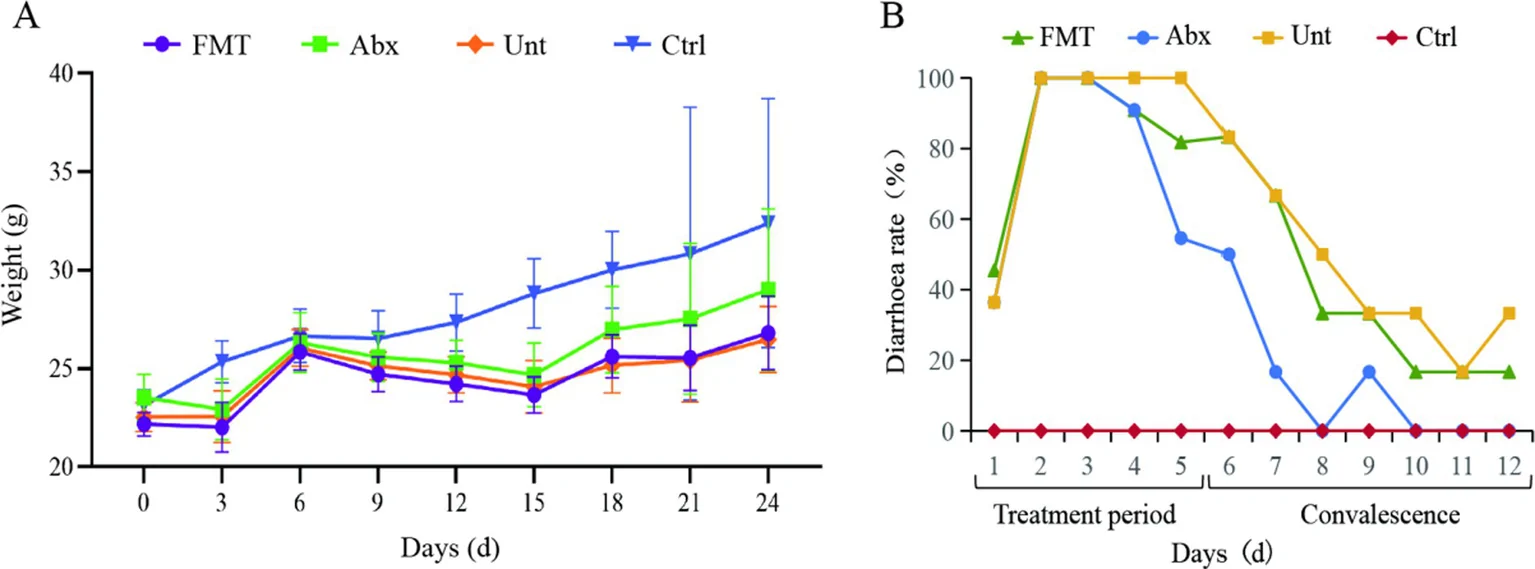
Body weight changes and diarrhea recovery in mice infected with MDR *S. enterica*. **(A)** Body weight changes in mice from FMT, Abx, Unt, and Ctrl. **(B)** Diarrhea rates in each group during the treatment and recovery phases.

All infected groups exhibited weight loss during the early infection phase, followed by gradual recovery (Figure 2A). The Abx group showed the highest weight gain (+5.5 g), followed by the FMT group (+4.7 g), while the Unt group gained the least (+3.9 g). The healthy Ctrl group maintained the highest overall gain (+9.3 g; Supplementary Table S9).

Diarrhea resolution followed a similar trend (Figure 2B). Diarrhea rates in the FMT and Abx groups began to decline on day 3 of treatment, whereas the Unt group maintained a high rate until day 7. By day 10, diarrhea had completely resolved in the Abx group and partially in the FMT group, while the Unt group showed incomplete recovery. No diarrhea was observed in the Ctrl group.

### 3.3 Histopathological evaluation of infection-induced tissue injury and therapeutic effects

**Figure 3:**
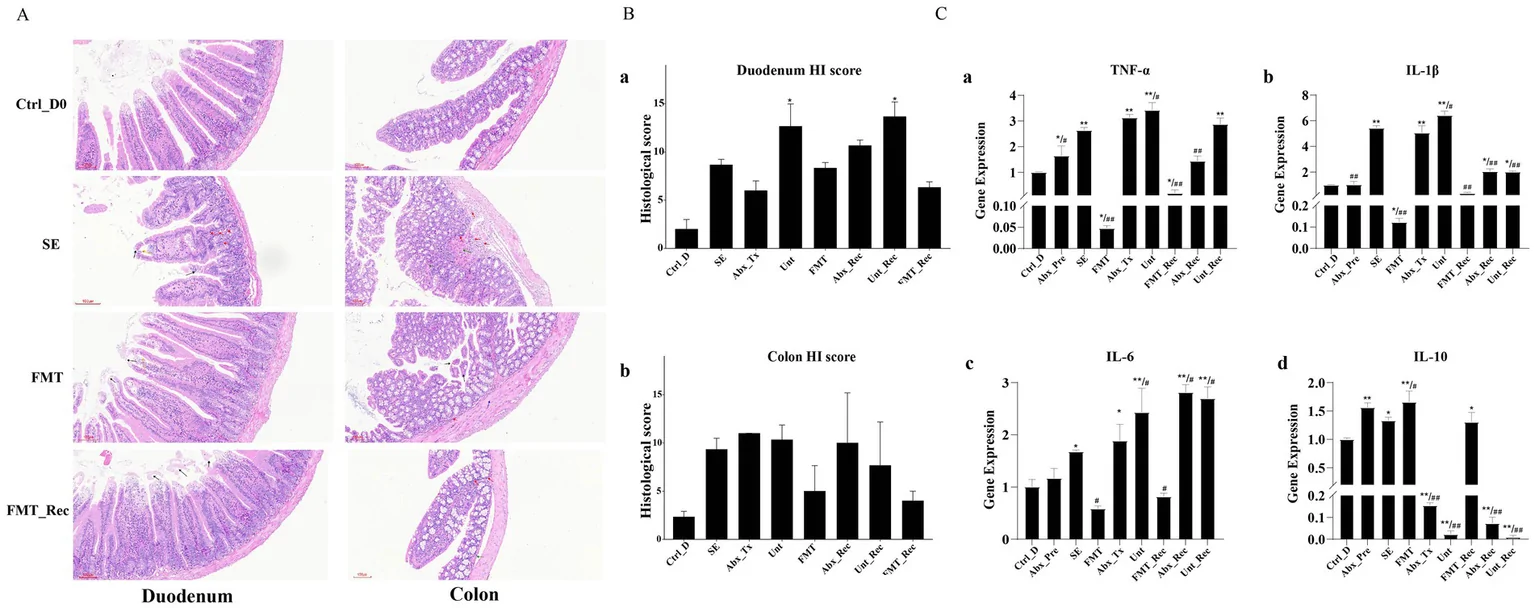
Histopathological evaluation and inflammatory gene expression following FMT treatment. **(A)** Representative H&E-stained sections of duodenum (left) and colon (right) from Ctrl_D0, SE, FMT, and FMT_Rec. Scale bar = 100 μm. **(B)** Histopathological scores of duodenum and colon tissues (*n* = 3/group). Duodenal injury was evaluated based on villus integrity, crypt architecture, epithelial integrity, inflammatory cell infiltration, and mucosal edema/congestion. Colonic injury was evaluated based on mucosal structural integrity, crypt architecture, epithelial integrity, inflammatory cell infiltration, and mucosal edema/congestion. Each parameter was scored from 0 to 4 according to predefined criteria, with higher scores indicating more severe injury.*p < 0.05, **p < 0.001 **(C)** Relative mRNA expression of colonic inflammatory genes, including *Tnf*, *Il1b*, *Il6*, and *Il10*. Data are presented as mean ± ± SD. *p < 0.05, **p < 0.001, ***p < 0.001. vs. Ctrl_D; ^#^*p* < 0.05, *##p* < 0.001 vs. SE.

Histological examination revealed intestinal injury following *S. enterica* infection. In the duodenum, infected mice exhibited villus shortening, epithelial disruption, and inflammatory cell infiltration. Consistently, histopathological scoring showed increased injury scores in SE groups compared with healthy controls. FMT-treated mice exhibited reduced duodenal histopathological scores and improved mucosal morphology, although the scores remained higher than those of control mice (Figures 3A,B; Supplementary Figure S1).

In the colon, *S. enterica* infection resulted in epithelial damage, crypt disruption, and inflammatory cell infiltration. Compared with untreated infected mice, FMT treatment was associated with reduced histopathological severity and improved epithelial integrity during the recovery phase. Antibiotic treatment also showed partial improvement but did not completely normalize colonic architecture (Figures 3A,B; Supplementary Figure S1). Cecal tissues showed extensive epithelial necrosis, lamina propria inflammation, and tissue edema following infection. FMT-treated mice showed reduced inflammatory damage and improved epithelial architecture by day 7 of recovery. Antibiotic treatment yielded partial improvement but failed to fully resolve epithelial disruption (Supplementary Figure S1).

### 3.4 FMT modulates inflammation-associated gene expression during MDR *Salmonella enterica* infection

To further characterize inflammatory responses associated with these histological improvements, the transcriptional expression of key cytokine genes in colonic tissues was analyzed (Figure 3C). Compared with the Ctrl_D, the SE group exhibited significantly increased mRNA expression of *Tnf*, *Il6*, and *Il1b* (*p* < 0.001) and decreased *Il10* expression (*p* < 0.05), confirming successful model establishment. In the FMT-treated groups (FMT and FMT_Rec), the mRNA expression levels of *Tnf*, *Il6*, and *Il1b* were lower than those in the SE group (*p* < 0.05), while *Il10* expression was higher (*p* < 0.05). This pattern was maintained during the recovery phase. In the antibiotic-treated groups (Abx_Tx and Abx_Rec) and untreated groups (Unt and Unt_Rec), *Tnf* and *Il6* expression levels remained elevated, and *Il10* expression remained lower than in the control group. The Abx_Pre group showed increased *Tnf* and *Il6* expression and decreased *Il10* expression compared with the control group (*p* < 0.05) (Figure 3C). Overall, FMT treatment was associated with reduced expression of pro-inflammatory cytokine genes and increased *Il10* transcription compared with the SE group.

### 3.5 FMT restores microbial diversity and beneficial taxa in *Salmonella*-infected mice

**Figure 4:**
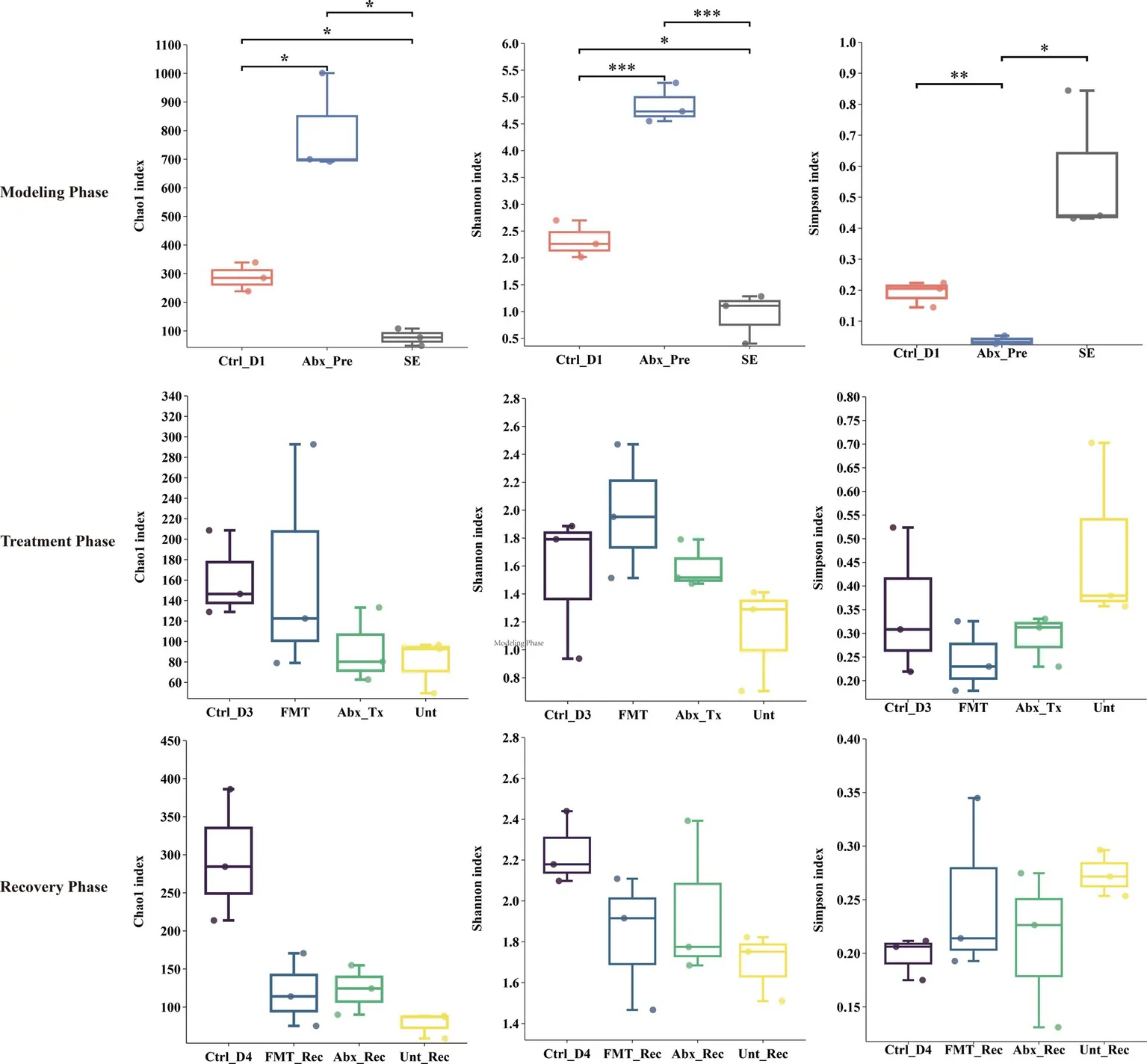
Alpha diversity of the small intestinal microbiota across experimental phases. Modeling phase; treatment phase; recovery phase. Chao1 richness, Shannon diversity, and Simpson index are shown for the indicated groups. Data are presented as mean ± SD (or median with interquartile range where appropriate). Each dot represents one biological sample. For microbiota analysis, three independent biological samples per group were analyzed (*n* = 3/group). Statistical significance among groups was assessed by Kruskal–Wallis tests followed by Dunn’s *post hoc* analysis with Benjamini–Hochberg correction. *p < 0.05, *p < 0.01, *p < 0.001.

Alpha diversity was evaluated across the modeling, treatment, and recovery phases (Figure 4; Supplementary Tables S10, S11). During the modeling phase, the SE group showed lower Chao1, Shannon, and Pielou indices compared with the control group (Dunn-BH, *p* < 0.05; Figure 4). In contrast, the Abx_Pre group exhibited higher Chao1 and Shannon indices and lower Simpson index relative to controls. Differences among groups were consistently observed across multiple diversity indices. During the treatment phase, Chao1 richness was lower in the Unt group than in the control group (*p* < 0.05). The FMT and Abx_Tx groups showed Chao1 values comparable to the control group. Shannon and Simpson indices remained similar across all groups, indicating no detectable differences in community evenness during this phase. During the recovery phase, Chao1 richness remained lower in all treatment groups compared with the control group (*p* < 0.05). In contrast, Shannon and Simpson indices were comparable among groups. Effect size values and corresponding confidence intervals for each comparison are provided in Supplementary Table S11.

**Figure 5:**
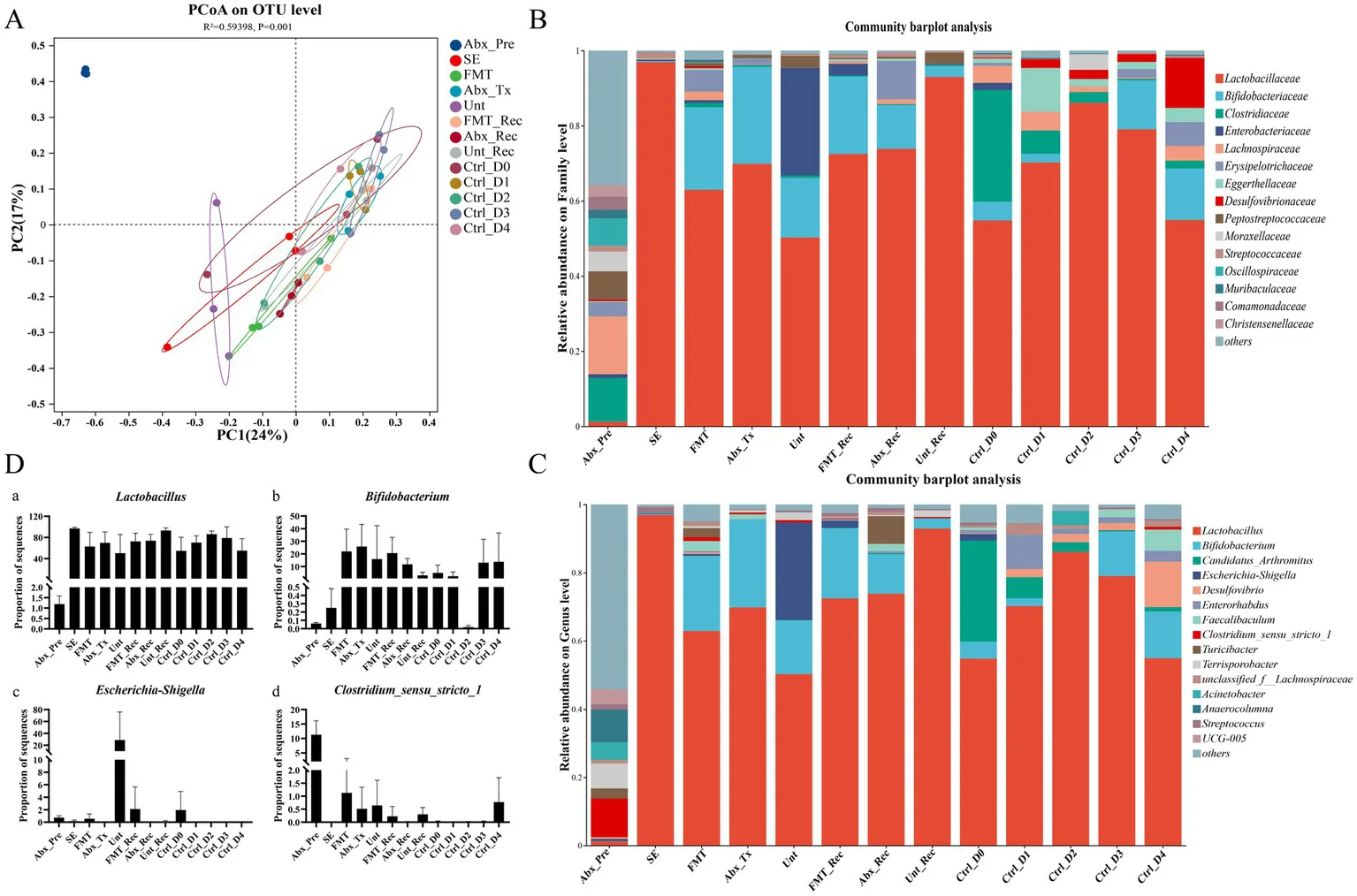
Composition and beta diversity of the small-intestinal microbiota in mice from different experimental groups. **(A)** Principal coordinate analysis (PCoA) of intestinal microbiota beta diversity at the OTU level. Each point in the PCoA plot represents one biological sample. **(B)** Relative abundance of the intestinal microbiota at the family level. **(C)** Relative abundance of the intestinal microbiota at the genus level. **(D)** Relative abundance of *Lactobacillus*, *Bifidobacterium*, *Escherichia-Shigella*, and *Clostridium_sensu_stricto_1*.

Beta diversity analysis based on principal coordinate analysis (PCoA) revealed distinct clustering patterns among experimental groups (Figure 5A). The SE and Unt groups were separated from the control group along the primary coordinate axis, whereas the FMT and FMT_Rec groups were located closer to the control cluster. The Abx_Tx group showed intermediate positioning between infected and control groups.

At the family and genus levels (Figures 5B–D), the SE group showed higher relative abundance of *Escherichia-Shigella* and *Enterococcus* compared with the control group. In contrast, the FMT_Rec group showed higher relative abundance of *Lactobacillus* and *Bifidobacterium*. The Unt_Rec group was characterized by higher relative abundance of *Streptococcus* and *Romboutsia*. The relative abundance of *Clostridium_sensu_stricto_1* varied across groups without a consistent directional pattern.

**Figure 6:**
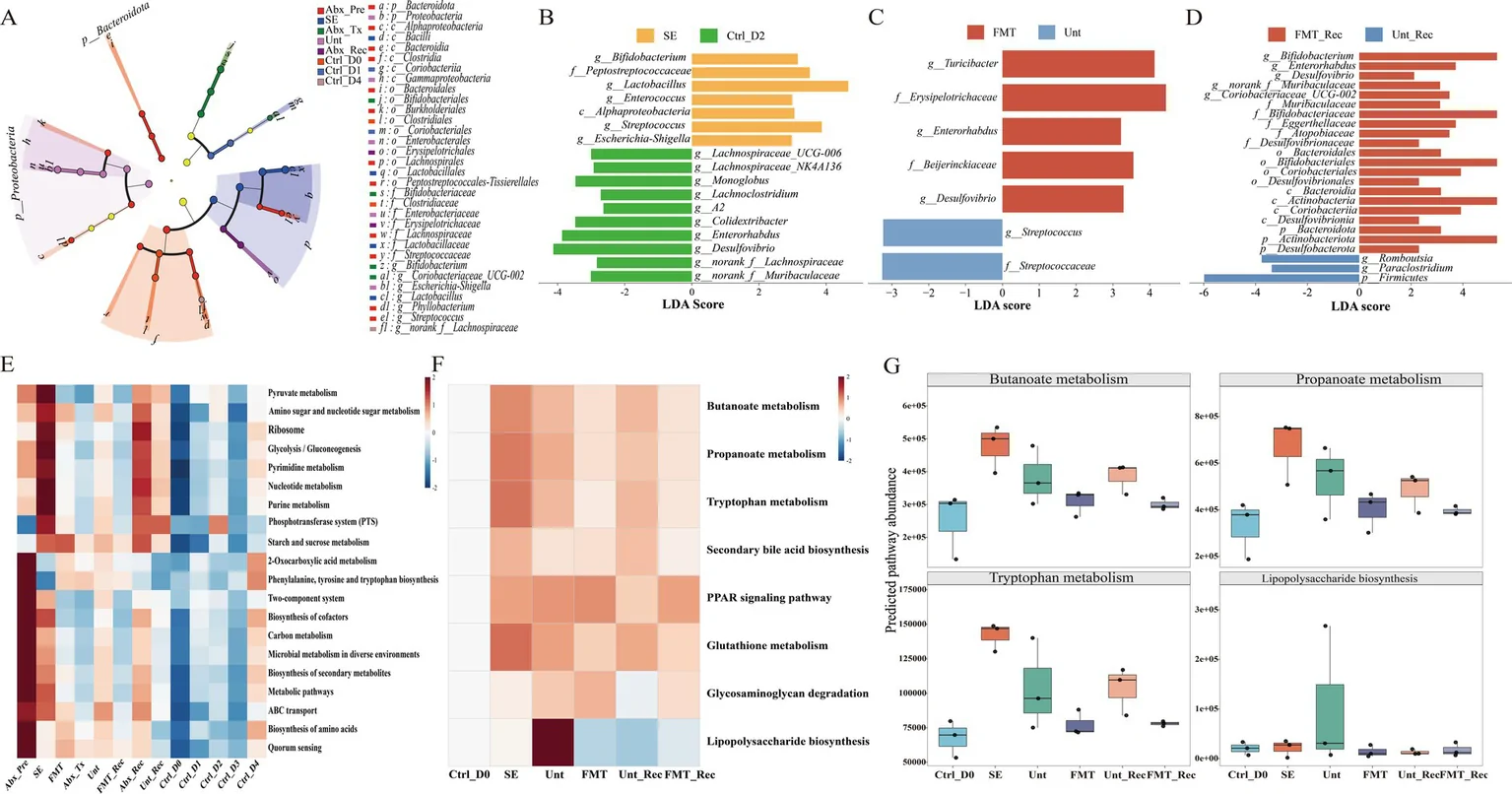
LEfSe and PICRUSt2 analysis of the small-intestinal microbiota. **(A)** LEfSe-derived cladogram illustrating differentially abundant taxa from phylum to genus level across all experimental groups. Node size is proportional to relative abundance; colors indicate the group in which taxa are significantly enriched. **(B–D)** Differentially abundant taxa identified by LEfSe (LDA score > 2.0, *p* < 0.05): **(B)** SE vs. Ctrl_D2, **(C)** FMT vs. Unt, **(D)** FMT_Rec vs. Unt_Rec. **(E,F)** PICRUSt2-predicted KEGG pathway heatmaps. **(G)** Predicted abundance of butanoate metabolism, propanoate metabolism, tryptophan metabolism, and lipopolysaccharide biosynthesis; values represent numerical trends without statistically significant differences among groups.

LEfSe analysis (LDA score > 2.0, *p* < 0.05) identified differentially abundant taxa among groups (Figures 6A–D). The SE group was enriched in *Enterococcus* and *Escherichia-Shigella*, whereas the FMT_Rec group was enriched in *Bifidobacterium* and Muribaculaceae. The Unt_Rec group showed enrichment of *Streptococcus* and *Romboutsia*. Desulfovibrio was also identified as an enriched taxon in the FMT_Rec group.

PICRUSt2-based functional prediction identified no statistically significant differences in predicted KEGG pathway abundances among the experimental groups (all adjusted *p* > 0.05; Figures 6E–G). Predicted pathways including lipopolysaccharide biosynthesis, butanoate metabolism, and propanoate metabolism showed numerical differences across groups; however, these patterns did not reach statistical significance and were therefore considered exploratory. Overall, FMT treatment was associated with changes in small-intestinal microbial diversity, community structure, and taxonomic composition compared with the infected and untreated groups.

### 3.6 FMT promotes recovery of colonic microbiota diversity and composition after *Salmonella enterica* infection

**Figure 7:**
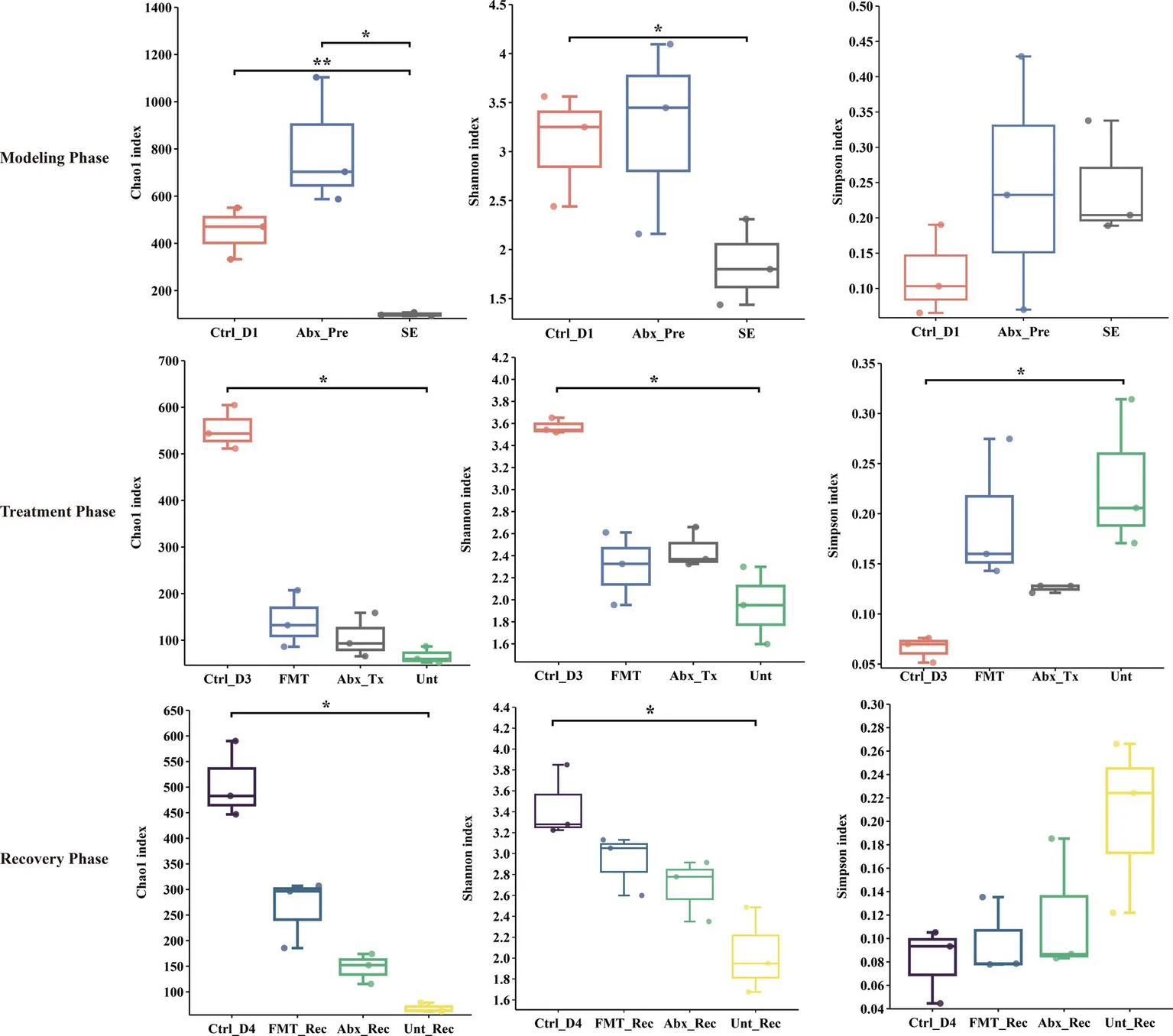
Alpha diversity of the colon microbiota across experimental phases. Modeling phase; treatment phase; recovery phase. Chao1 richness, Shannon diversity, and Simpson index are shown for the indicated groups. Data are presented as mean ± SD (or median with interquartile range where appropriate). Each dot represents one biological sample. For microbiota analysis, three independent biological samples per group were analyzed (*n* = 3/group). Statistical significance among groups was assessed by Kruskal–Wallis tests followed by Dunn’s *post hoc* analysis with Benjamini-Hochberg correction. *p < 0.05, *p < 0.01, *p < 0.001.

Alpha diversity was analyzed across modeling, treatment, and recovery phases (Figure 7; Supplementary Tables S12, S13). During the modeling phase, Abx_Pre resulted in exceptionally high richness and evenness (Simpson index: 0.04 ± 0.01), suggesting dysbiotic expansion. In contrast, SE significantly reduced Chao1, Shannon, and Pielou indices compared with controls (Dunn-BH, *p* < 0.05), with large effect sizes (Cohen’s d < −2.4; Supplementary Table S13). During the treatment phase, Shannon and Simpson indices were comparable among FMT, Abx, and Unt groups (*p* > 0.05), although the Unt group showed significantly lower Chao1 richness than controls. By the recovery phase, Shannon and Simpson indices recovered to control levels across all treatment groups (*p* > 0.05), whereas Chao1 richness remained significantly lower than controls. Effect size analysis indicated that untreated mice still exhibited large negative effects versus controls, while FMT showed attenuated effects with confidence intervals overlapping zero (Supplementary Table S13).

**Figure 8:**
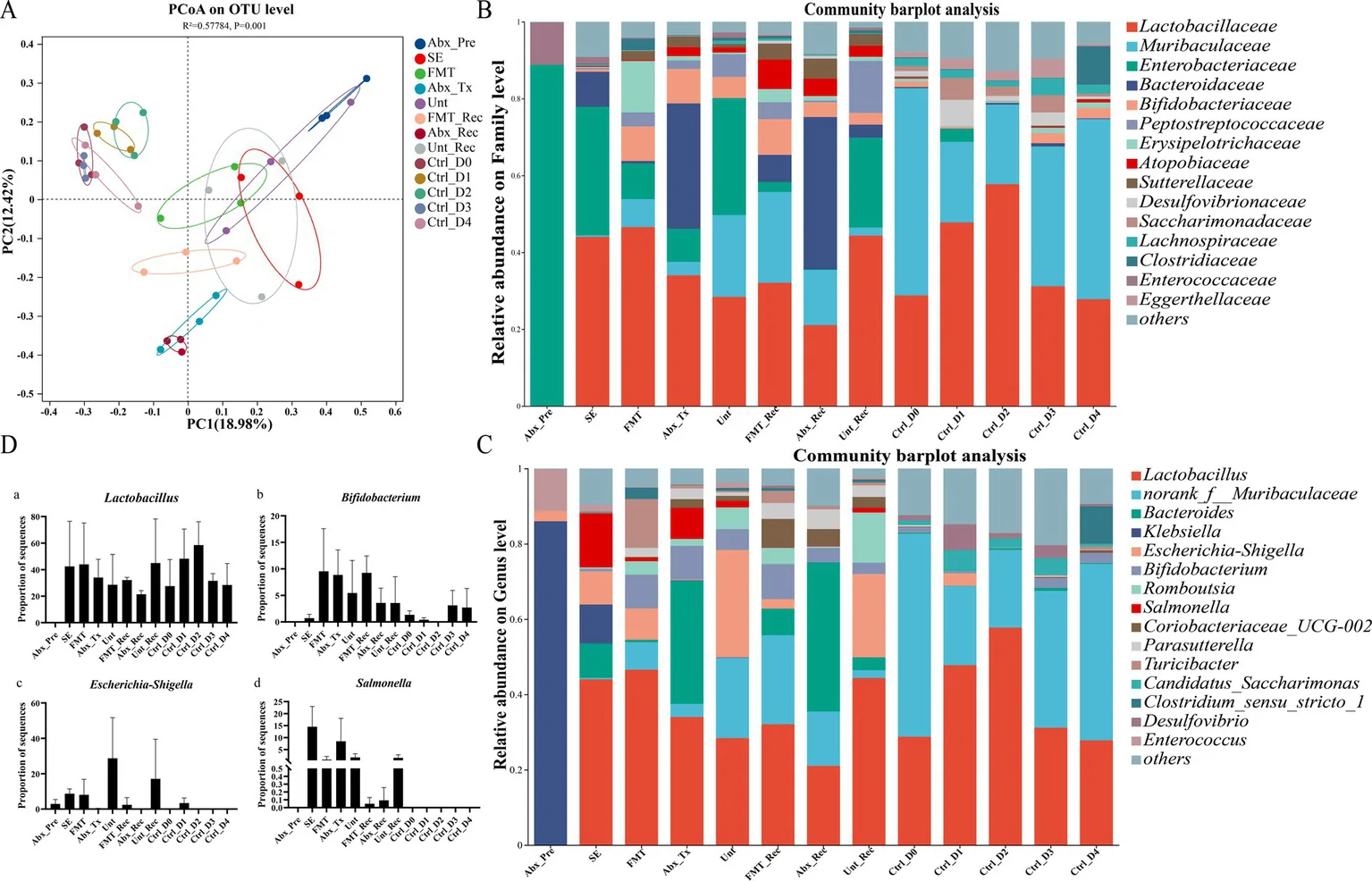
Composition and beta diversity of the colonic microbiota in mice from different experimental groups **(A)** Principal coordinate analysis (PCoA) of colonic microbiota beta diversity at the OTU level. Each point in the PCoA plot represents one biological sample. **(B)** Relative abundance of the colonic microbiota at the family level. **(C)** Relative abundance of the colonic microbiota at the genus level. **(D)** Relative abundance of *Lactobacillus*, *Bifidobacterium*, *Escherichia*-*Shigella*, and *Clostridium* sens*u stricto 1*.

PCoA at the OTU level revealed distinct clustering among groups (Figure 8A). The FMT and FMT_Rec groups shifted markedly toward the control group, indicating restoration of colonic *β*-diversity. At the family and genus levels (Figures 8B–D), *Salmonella* infection led to reduced Lactobacillaceae and Bifidobacteriaceae, and increased Enterobacteriaceae and *Escherichia-Shigella*. FMT treatment reversed these alterations: *Lactobacillus* and *Bifidobacterium* increased, while *Escherichia-Shigella* and *Salmonella* decreased. The abundance of *Clostridium_sensu_stricto_1* tended to return toward control levels.

**Figure 9:**
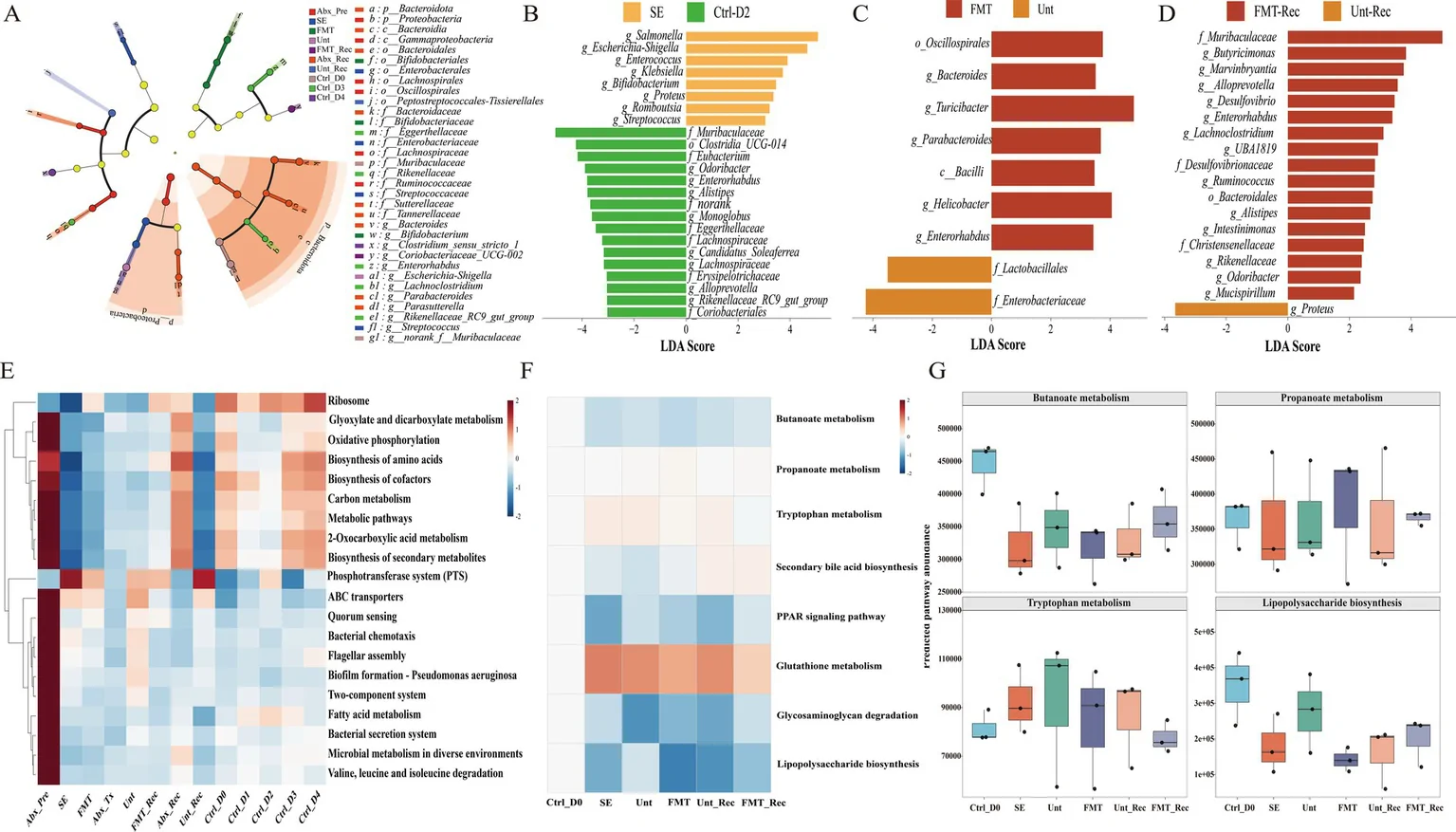
LEfSe and PICRUSt2 analysis of the colon microbiota. **(A)** LEfSe-derived cladogram illustrating differentially abundant taxa from phylum to genus level across all experimental groups. Node size is proportional to relative abundance; colors indicate the group in which taxa are significantly enriched. **(B–D)** Differentially abundant taxa identified by LEfSe (LDA score > 2.0, *p* < 0.05): **(B)** SE vs. Ctrl_D2, **(C)** FMT vs. Unt, **(D)** FMT_Rec vs. Unt_Rec. **(E,F)** PICRUSt2-predicted KEGG pathway heatmaps. **(G)** Predicted abundance of butanoate metabolism, propanoate metabolism, tryptophan metabolism, and lipopolysaccharide biosynthesis; values represent numerical trends without statistically significant differences among groups.

LEfSe analysis (LDA score > 2.0, *p* < 0.05) identified differentially abundant taxa among groups (Figures 9A–D). During the infection phase, the SE group was enriched in *Salmonella*, *Escherichia-Shigella*, *Klebsiella*, and *Proteus*, whereas the Ctrl_D2 group was enriched in Muribaculaceae, Lachnospiraceae, and *Rikenellaceae*. Following treatment, the FMT group was enriched in *Oscillospirales*, *Bacteroides*, and *Turicibacter*, whereas the Unt group remained enriched in Enterobacteriaceae-related taxa. During the recovery phase, the FMT_Rec group was enriched in Muribaculaceae, *Butyricimonas*, and *Marvinbryantia*, while the Unt_Rec group remained enriched in *Proteus*.

PICRUSt2-based functional prediction identified no statistically significant differences in predicted KEGG pathway abundances among the experimental groups (all adjusted *p* > 0.05; Figures 9E–G). Predicted pathways including lipopolysaccharide biosynthesis, butanoate metabolism, propanoate metabolism, and tryptophan metabolism showed numerical differences across groups; however, these patterns did not reach statistical significance and were therefore considered exploratory. Overall, FMT treatment was associated with changes in colonic microbial diversity and taxonomic composition compared with the infected and untreated groups.

### 3.7 FMT treatment was associated with loss of IncHI2A-associated resistance determinants in recovered *Salmonella* isolates

**Figure 10:**
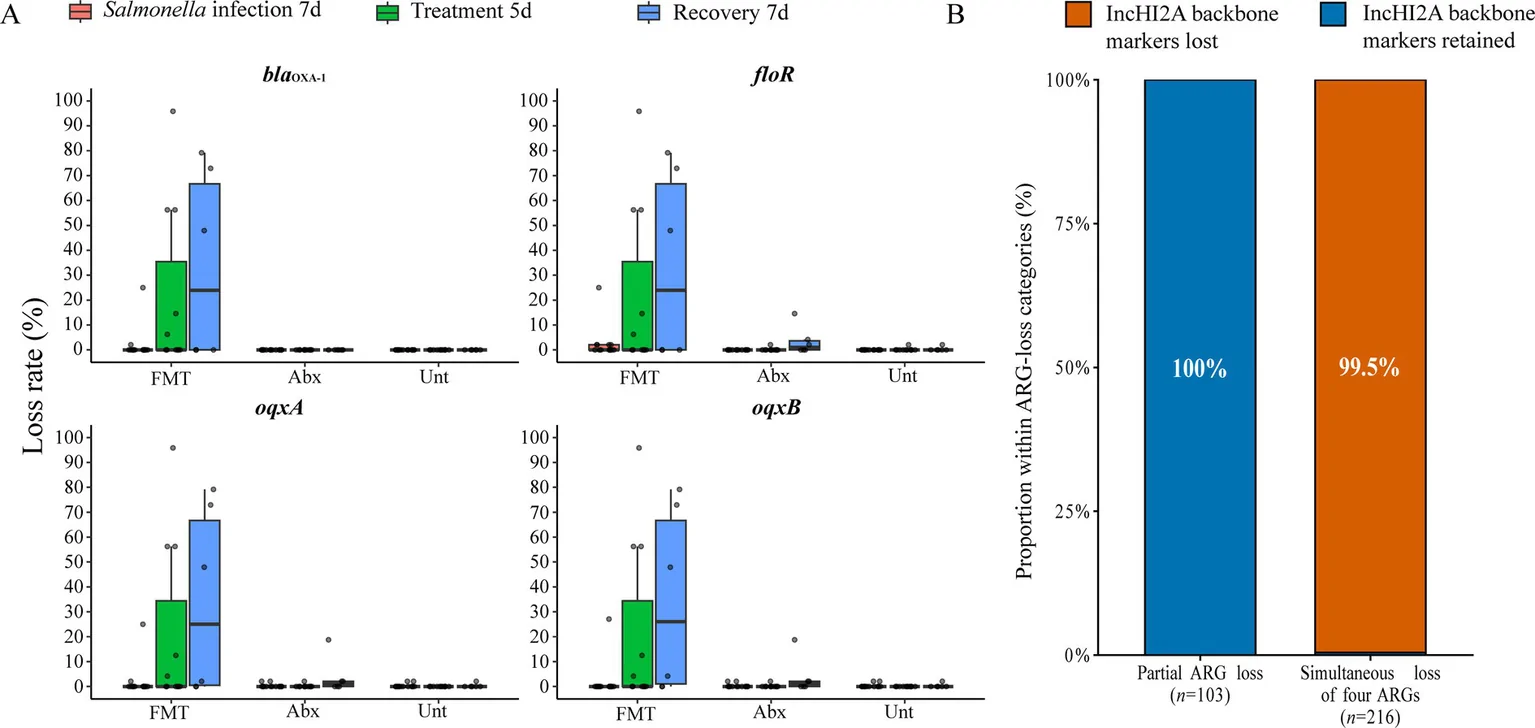
Association between FMT treatment and IncHI2A-associated resistance determinant instability in recovered *Salmonella* isolates. **(A)** Longitudinal detection patterns of four IncHI2A-associated resistance genes (*bla*_OXA-1_, *floR*, *oqxA*, and *oqxB*) in *Salmonella* isolates from FMT, Abx, and Unt groups. Forty-eight colonies per sample were screened at each sampling time point. Forty-eight colonies were screened per mouse at each sampling time point. The numbers of mice evaluated per group were *n* = 13 during the infection phase, *n* = 11 at the end of treatment, and *n* = 6 during recovery. **(B)** Association between resistance determinant loss patterns and detectable IncHI2A-associated backbone markers. Among isolates showing simultaneous loss of four resistance determinants (*n* = 216), 215/216 (99.5%) exhibited loss of detectable *repB* and *parB*-associated regions, whereas one isolate retained detectable backbone markers. In contrast, isolates showing partial resistance determinant loss (*n* = 103) retained both detectable *repB* and *parB* markers.

Whole-genome sequencing confirmed that *bla*_OXA-1_, *floR*, *oqxA*, and *oqxB* were co-localized on a single IncHI2A plasmid (pCP174-*bla*_OXA-1_) (Figure 1). To investigate the dynamics of IncHI2A-associated resistance determinants following FMT treatment, the four resistance genes were longitudinally monitored in *Salmonella* isolates recovered from the FMT, Abx, and Unt groups (Figure 10). To further explore the genetic basis underlying ARG loss, all ARG-loss isolates were screened for the presence of *repB* and *parB*, two IncHI2A-associated backbone markers.

In the FMT group, resistance gene loss increased progressively over time. At day 7 post-infection, loss was infrequent: only 5 of 13 mice (38.5%) exhibited detectable loss, with individual gene loss rates ranging from 2.08 to 27.08% (Figure 10). By the end of the treatment phase, the proportion of mice showing ARG loss rose to 5 of 11 (45.5%), with two mice demonstrating simultaneous loss of all four genes at 56.25 and 95.83%, respectively. The remaining three mice exhibited partial co-loss patterns, primarily involving *floR* and *bla*_OXA-1_ (6.25%–14.58%) together with *oqxA* and *oqxB* (4.17%–12.50%). By day 7 of recovery (day 19), high loss rates were consistently observed: 3 of 6 mice (50.0%) showed concurrent loss of all four genes at rates between 47.92% and 79.17% (Figure 10).

In contrast, resistance gene loss remained minimal in both the Abx and Unt groups throughout the experiment. In the Abx group, sporadic, low-level loss of *oqxA*/*oqxB* or *floR* was detected in only a few mice per time point, always below 20.0%, and no *bla*_OXA-1_ loss was observed. In the Unt group, loss rates did not exceed 2.08% at any time point, with the majority of mice showing no detectable loss (Figure 10).

To determine whether the observed ARG loss resulted from spontaneous instability during bacterial propagation, the original P174 strain was subjected to 19 serial passages under non-selective *in vitro* conditions. At generations 7, 12, and 19, 48 individual colonies were randomly selected and screened for the four ARGs and IncHI2A backbone markers (*repB* and *parB*). No loss of *bla*_OXA-1_, *floR*, *oqxA*, *oqxB*, *repB*, or *parB* was detected throughout serial passage, indicating that the ARG loss and backbone marker loss observed *in vivo* were unlikely to result from spontaneous loss under laboratory conditions during routine laboratory propagation.

Phenotypic validation by MIC testing revealed reduced resistance to ampicillin, florfenicol, ciprofloxacin, and enrofloxacin in isolates that had lost the corresponding genes. PCR confirmation further demonstrated that >95% of isolates with reduced MICs lacked the target resistance genes. Across all ARG-loss isolates recovered from the FMT, Abx, and Unt groups, 216 of 319 isolates (67.7%) exhibited simultaneous loss of *bla*_OXA-1_, *floR*, *oqxA*, and o*qxB*, whereas 103 isolates (32.3%) showed partial loss patterns. Among the 216 isolates with simultaneous four-gene loss, 215 lacked both *repB* and *parB*, whereas one isolate retained both backbone markers. In contrast, all 103 isolates with partial ARG loss retained *repB* and *parB*. These findings indicate that loss of detectable IncHI2A-associated backbone markers was strongly associated with simultaneous loss of multiple resistance determinants, whereas partial ARG loss may result from localized deletion or rearrangement of resistance-associated regions.

## 4 Discussion

This study showed that FMT alleviated MDR *S. enterica*-induced diarrhea in mice. FMT also reduced intestinal tissue injury, reshaped the gut microbiota, and was associated with increased loss of IncHI2A plasmid-associated resistance genes in *Salmonella* isolates. These findings suggest that FMT may protect the host through several related mechanisms. These include restoration of microbial ecology, improvement of epithelial barrier integrity, regulation of mucosal inflammation, and reduction of plasmid-associated antimicrobial resistance determinants.

After establishing a murine model of MDR *S. enterica*-induced diarrhea, we observed that FMT promoted body weight recovery and reduced diarrheal symptoms. This is consistent with previous reports (Zhang et al., 2020). In addition to symptom improvement, *Salmonella* abundance decreased in FMT-treated mice during the later phase of the experiment. This decrease may be related to the recovery of colonization resistance. Commensal microbiota can restrict enteric pathogens through nutrient competition, niche occupation, production of inhibitory metabolites, and regulation of host immunity (Ducarmon et al., 2019; Kumariya et al., 2019; Caballero-Flores et al., 2023). Therefore, the clinical improvement after FMT may reflect both pathogen restriction and recovery of the intestinal ecological barrier.

Histopathological analysis showed that FMT-treated mice had less epithelial disruption, villus shortening, crypt damage, and inflammatory cell infiltration than infected untreated mice. Restoration of epithelial barrier integrity is important for limiting bacterial translocation and reducing downstream activation of pro-inflammatory pathways (Ménard et al., 2010). Consistent with the histological findings, FMT was associated with decreased transcriptional expression of *Tnf*, *Il1b*, and *Il6* and increased *Il10* transcription in colonic tissues. IL-10 is a key anti-inflammatory cytokine produced by several immune cell populations. It plays an important role in limiting excessive mucosal inflammation (Burrello et al., 2018; Ouyang and O'Garra, 2019; Yang et al., 2023). TNF-α, IL-6, and IL-1β are not only inflammatory markers, but also mediators of epithelial injury and barrier dysfunction (Bischoff et al., 1999; Sommer et al., 2014; Zhang et al., 2022; Meyer et al., 2023). Thus, the cytokine profile observed in FMT-treated mice supports an immunomodulatory role of FMT in MDR *S. enterica*-induced intestinal inflammation. However, the present study evaluated inflammatory responses primarily at the transcriptional level. Although changes in cytokine gene expression were consistent with the observed histopathological improvement, mRNA abundance does not always directly reflect cytokine protein production or biological activity. Future studies incorporating protein-level analyses, such as ELISA or multiplex cytokine profiling, will further clarify the immunological mechanisms involved in FMT-mediated protection.

FMT-mediated microbial recovery differed between intestinal regions. *Salmonella* infection reduced microbial richness and diversity in both the small intestine and colon. This indicates broad ecological disruption. Antibiotic pretreatment increased richness but reduced evenness. This suggests the formation of an imbalanced microbial community. Antibiotics can deplete dominant commensal bacteria and reduce colonization resistance. This may allow low-abundance, tolerant, or resistant taxa to expand into newly available niches (Dethlefsen et al., 2008; Fishbein et al., 2023). Therefore, higher richness does not necessarily indicate a healthier microbiota under disturbed conditions. After FMT, the colon showed partial recovery of microbial diversity and community structure. Shannon and Simpson indices approached control levels during the recovery phase. However, Chao1 richness remained lower than that of controls. This suggests that dominant community structure may recover earlier than full taxonomic richness. In contrast, recovery in the small intestine was limited. This may be related to the unique ecological features of the small intestine. These include lower microbial biomass, faster luminal transit, stronger exposure to bile acids and digestive secretions, and greater environmental fluctuation than in the colon (Kastl et al., 2020; Jensen et al., 2023). Thus, microbiota recovery after infection and antibiotic exposure is not uniform across the gut. It is a gradual and spatially dependent process.

Taxonomic changes further supported FMT-mediated ecological restructuring. FMT-treated mice showed lower abundance of Enterobacteriaceae-related taxa. They also showed higher abundance of *Lactobacillus*, *Bifidobacterium*, Muribaculaceae, Lachnospiraceae, and other anaerobic taxa. These changes are biologically meaningful. Recovery of anaerobic commensal bacteria is closely linked to colonization resistance against enteric pathogens (Buffie and Pamer, 2013; Khan et al., 2021). *Lactobacillus* and *Bifidobacterium* can produce organic acids and bacteriocin-like metabolites. Muribaculaceae and Lachnospiraceae are often associated with carbohydrate fermentation and short-chain fatty acid production (Duranti et al., 2016; Burrello et al., 2018; Li et al., 2024; Liu et al., 2024; Pujato et al., 2024). In this study, these microbial changes occurred together with reduced *Salmonella* abundance and lower inflammatory cytokine expression. This suggests that FMT-mediated microbiota restructuring may contribute to pathogen restriction and mucosal immune recovery.

PICRUSt2 analysis provided indirect information on potential microbial functional changes. No KEGG pathway showed a statistically significant difference among groups. Therefore, these predicted functional changes should be interpreted with caution. However, several pathways showed numerical variation. These included lipopolysaccharide biosynthesis, butanoate metabolism, propanoate metabolism, and tryptophan metabolism. These pathways are biologically relevant. Lipopolysaccharide is a major outer-membrane component of Gram-negative bacteria and can act as a strong inflammatory stimulus (Raetz and Whitfield, 2002). In contrast, butyrate, propionate, and tryptophan-derived metabolites help maintain epithelial barrier function. They also regulate mucosal immunity and contribute to host defense against intestinal pathogens (Agus et al., 2018; Parada Venegas et al., 2019). These biological roles provide context for the exploratory pathway patterns but should not be considered mechanistic evidence from the present study. Direct functional validation using metagenomic, metatranscriptomic, or metabolomic approaches will be required.

A key finding of this study was the increased loss of IncHI2A plasmid-associated ARGs in *Salmonella* isolates from FMT-treated mice. Whole-genome sequencing confirmed that *bla*_OXA-1_, *floR*, *oqxA*, and *oqxB* were co-localized on the IncHI2A plasmid pCP174-*bla*_OXA-1_. In the FMT group, loss of these four genes increased during the treatment and recovery phases. In contrast, ARG loss was rare in antibiotic-treated and untreated mice. No ARG loss was detected after serial *in vitro* passaging. This indicates that ARG loss was associated with the *in vivo* intestinal environment, rather than spontaneous instability during laboratory propagation.

Previous clinical studies have provided encouraging but heterogeneous evidence regarding FMT-mediated decolonization of multidrug-resistant organisms. In a randomized clinical trial, Huttner et al. reported clearance of extended-spectrum β-lactamase-producing or carbapenemase-producing Enterobacteriaceae in 41% of patients receiving non-absorbable antibiotics followed by FMT, compared with 29% in the untreated control group; however, the difference was not statistically conclusive (Huttner et al., 2019). In a prospective study of 20 carriers of carbapenemase-producing Enterobacterales, Davido et al. observed complete clearance in 20% of recipients at 2 weeks and 40% at 3 months after FMT. Successful clearance was associated with greater post-FMT bacterial richness and diversity, indicating that the ecological response to FMT may influence decolonization outcomes (Davido et al., 2024). More recently, the FERARO randomized feasibility trial showed that repeated encapsulated FMT was feasible, acceptable, and generally well tolerated in patients carrying extended-spectrum β-lactamase-producing or carbapenem-resistant Enterobacterales. Although the trial was not powered to establish efficacy, greater donor-strain engraftment tended to be associated with successful decolonization (Merrick et al., 2025). Together with the reductions in MDRO carriage and antimicrobial resistance burden reported by Woodworth et al. (2023) and Nooij et al. (2024), these studies support the potential of microbiota restoration as a strategy for modifying the intestinal reservoir of antimicrobial resistance (Woodworth et al., 2023; Nooij et al., 2024). However, the variable decolonization rates also indicate that treatment outcomes may depend on the recipient microbiota, donor engraftment, baseline pathogen burden, concomitant antibiotic exposure, and the FMT regimen.

The present study complements these clinical investigations but addresses a different biological level. Previous studies have predominantly evaluated culture-defined MDRO carriage, pathogen abundance, or changes in the overall gut resistome. In contrast, we examined resistance determinant dynamics directly within *Salmonella* isolates recovered from individual animals. Thus, the association between FMT and increased loss of IncHI2A-associated resistance determinants provides pathogen-level evidence that microbiota restoration may influence not only MDR pathogen persistence but also the stability of resistance-associated genetic elements within the pathogen population. Nevertheless, these data do not demonstrate that individual microbial taxa or metabolites directly caused resistance determinant loss. FMT may instead modify several interacting ecological factors, including nutrient and niche competition, microbiota-derived metabolites, pathogen population structure, and the fitness costs associated with maintaining resistance elements in the absence of corresponding antibiotic selection.

Among ARG-loss isolates recovered from different experimental groups, 67.7% simultaneously lost all four tested ARGs. Additional PCR analysis showed that isolates exhibiting simultaneous loss of *bla*_OXA-1_, *floR*, *oqxA*, and *oqxB* also lacked the IncHI2A backbone markers *repB* and *parB*, supporting the involvement of IncHI2A-associated genetic element loss in this co-loss pattern. However, the presence of *repB* and *parB* in some ARG-loss isolates indicates that resistance determinants can also be removed independently of complete backbone loss, possibly through deletion or rearrangement of resistance-associated regions. However, partial gene-loss patterns were also observed. This suggests that more than one genetic process may have occurred. Plasmids and other mobile genetic elements are important vehicles for ARG maintenance and spread in complex microbial communities (Castañeda-Barba et al., 2024). Therefore, future studies should use long-read sequencing, plasmid reconstruction, and plasmid stability assays. These approaches will help clarify the genetic basis of complete and partial ARG loss. Overall, these findings suggest that FMT helps restore a healthy gut environment. As the gut microbiota recovers, Enterobacteriaceae-related bacteria become less dominant. This changed environment may reduce the selective advantage and stability of maintaining IncHI2A-associated resistance elements. Therefore, FMT-induced ARG loss should not be interpreted as simple plasmid curing. Instead, restoration of the gut microbial ecosystem may alter the ecological conditions that influence the maintenance and stability of IncHI2A-associated resistance elements. Instead, it may work in several ways: *Salmonella* strains that lose the plasmid may grow better in the restored gut; the plasmid itself may become less stable without strong antibiotic pressure; or FMT may simply reduce the overall advantage of carrying resistance genes when antibiotics are no longer present.

Several limitations should be acknowledged. First, the structural basis of ARG loss remains unresolved because representative ARG-loss isolates were not subjected to long-read sequencing. Second, 16S rRNA sequencing provides mainly taxonomic information and cannot identify mobile-element hosts, quantify horizontal gene transfer, or directly assess microbial function. The small number of animals included in microbiota profiling also limited statistical power. Third, PICRUSt2 revealed no significant pathway differences after multiple-testing correction; therefore, the observed numerical trends should not be interpreted as mechanistic evidence. In addition, microbiota analysis was performed using three randomly selected biological replicates per group. Although this design enabled characterization of major microbial community shifts following FMT treatment, the relatively small sample size may limit statistical power. Therefore, the identified microbial alterations and predicted functional changes should be interpreted cautiously and require confirmation in larger-scale studies. A limitation of this study is that donor microbiota composition was not independently characterized, and broader microbiological screening beyond the predefined Salmonella and resistance determinant targets was not performed. These factors may influence donor variability, FMT reproducibility, and safety assessment.

Translation of these findings also requires caution. Differences among mice, humans, livestock, and companion animals in gut microbiota, intestinal physiology, immune function, diet, husbandry, and *Salmonella* epidemiology may limit direct extrapolation (Ericsson and Franklin, 2021). In addition, FMT outcomes depend on donor screening, preparation, dose, administration route, treatment frequency, and recipient characteristics, and inadequate screening may introduce pathogens or resistance determinants (Mullish et al., 2024; Winston et al., 2024). Therefore, the present findings should be considered preclinical. Future studies should combine long-read sequencing, metagenomic and metabolomic analyses, larger animal cohorts, naturally infected veterinary models, and controlled clinical studies to evaluate the safety, durability, and generalizability of FMT for controlling MDR *S. enterica.*

## 5 Conclusion

This study demonstrated that FMT alleviated diarrhea and intestinal injury caused by MDR *S. enterica* infection in mice. FMT was associated with partial restoration of gut microbial diversity, increased relative abundances of commensal anaerobic taxa, reduced *Salmonella* abundance, and attenuated transcriptional inflammatory responses. Importantly, *Salmonella* isolates recovered from FMT-treated mice showed increased loss of IncHI2A-associated resistance determinants and corresponding reductions in antimicrobial resistance phenotypes. These findings suggest that restoration of the gut microbiota may alter intestinal ecological conditions in ways that reduce the stability and persistence of IncHI2A-associated resistance elements. Overall, FMT represents a promising microbiota-based approach for controlling MDR *S. enterica* infection, although its efficacy, safety, and underlying mechanisms require further validation in larger and clinically relevant models.

## Data Availability

The whole-genome sequencing data of S. enterica strain P174 are available in the NCBI GenBank database under Nucleotide number JCBIYJ000000000 and BioProject accession PRJNA1502396. The processed 16S rRNA gene sequencing datasets are available on Figshare (DOI: 10.6084/m9.figshare.33121481).

## Footnotes

1
https://www.ncbi.nlm.nih.gov
