# Supplementary material for: FMT alleviates multidrug-resistant Salmonella enterica-induced diarrhea and is associated with loss of IncHI2A-associated resistance determinants in mice
Source: Front Microbiol. 2026 Aug 27;17:1913730. doi: 10.3389/fmicb.2026.1913730 (PMC13558225; doi:10.3389/fmicb.2026.1913730)
Supplement: Supplementary file 1 [file Supplementary_file_1.docx]

## Supplementary Table S1. Histopathological scoring criteria for intestinal injury evaluation.

| Parameter | 0 | 1 | 2 | 3 | 4 |
| --- | --- | --- | --- | --- | --- |
| Villus integrity (Duodenum) / mucosal structural integrity(Colon) | Normal architecture | Slight structural alteration or mild shortening | Moderate disruption with partial damage | Marked disruption with extensive damage | Severe destruction or loss of architecture |
| Crypt architecture | Normal crypt structure | Mild irregularity | Moderate crypt distortion or partial damage | Marked crypt disruption | Severe crypt destruction |
| Epithelial integrity | Intact epithelium | Rare focal epithelial detachment (single cells or small clusters, <5% of surface) | Epithelial disruption <25% | Epithelial disruption of 25–50% | Epithelial disruption >50% |
| Inflammatory cell infiltration | No obvious infiltration | Minimal infiltration | Moderate infiltration | Marked infiltration | Extensive diffuse infiltration |
| Mucosal edema/congestion | Absent | Mild edema or vascular dilation | Moderate edema or congestion | Severe edema with obvious congestion | Extensive edema and hemorrhagic changes |

Table S2 Primer sequences used for RT-qPCR(mouse derived genes) and plasmid PCR analyses

| Gene | Primer sequences (5' - 3') | Product ( bp ) | Purpose |
| --- | --- | --- | --- |
| *Il6* | F: TTGGGACTGATGCTGGTGA  R: TAGACAGGTCTGTTGGGAG | 95 | Mouse inflammatory gene expression |
| *Il10* | F: GTTGCCAAGCCTTATCG  R: CCGCATCCTGAGGGTCT | 131 | Mouse inflammatory gene expression |
| *Il1b* | F: GCAGCAGCACATCAACAAG  R: AGGTCCACGGGAAAGACAC | 95 | Mouse inflammatory gene expression |
| *Tnf* | F: TGCCACAAAGGAACCTACT  R: ATTCTGGGAAGCCGTAAA | 99 | Mouse inflammatory gene expression |
| *β-actin* | F: TCCAGCCTTCCTTCTTGGG  R: GTTGGCATAGAGGTCTTTACGG | 104 | Mouse inflammatory gene expression |
| *invA* | F: GTCCTCCGCCCTGTCTACT  R: TCCTAACGACGACCCTTC | 273 | *Salmonella* detection |
| *bla_OXA-1_* | F: ACCCGGAGCCTCATTAATTGTTAGC  R: GAATCTGGTGCCATTTGCGTTGC | 273 | ARG detection |
| *floR* | F: TGAACACGACGCCCGCTAT  R: GGACCGCTCCGCAAACAA | 750 | ARG detection |
| *oqxA* | F: CTCGGCGCGATGATGCT  R: CCACTCTTCACGGGAGACGA | 392 | ARG detection |
| *oqxB* | F: TTCTCCCCCGGCGGGAAGTAC  R: CTCGGCCATTTTGGCGCGTA | 513 | ARG detection |
| *repB* | F: GCGACCTTTCTTAACTTCTTGAT  R: ACCGAAACTTTGAAGTTGATGCC | 650 | IncHI2A backbone detection |
| *parB* | F:TTGTTGTATTCCCAGTGGCGTCC  R:CCACGCATATCCACTGTCCACCA | 317 | IncHI2A backbone detection |

Table S3 Genome assembly and quality assessment of *S. enterica* P174

| Parameter | Value |
| --- | --- |
| Number of contigs | 5 |
| Total assembly length | 5,085,986 bp |
| Largest contig | 3,215,400 bp |
| N50 | 3,215,400 bp |
| L50 | 1 |
| GC content | 52.01% |
| Completeness | 99.99% |
| Contamination | 0.19% |
| Predicted rRNA genes | 22 |

Table S4 Prediction of drug resistance information

| Drug resistance genes | Login number | Similarity（%） | Remarks |
| --- | --- | --- | --- |
| *aac(3)-IVa* | NG_047253.1 | 100.0 | aminoglycoside N-acetyltransferase AAC(3)-IVa |
| *aph(4)-Ia* | NG_047456.1 | 100.0 | aminoglycoside O-phosphotransferase APH(4)-Ia |
| *aac(6')-Ib-D181Y* | NG_067946.1 | 100.0 | *AAC(6')-Ib family aminoglycoside 6'-N-acetyltransferase* |
| *aadA1* | NG_052266.1 | 100.0 | *ANT(3'')-Ia family aminoglycoside nucleotidyltransferase AadA1* |
| *aadA2* | NG_051846.1 | 100.0 | *ANT(3'')-Ia family aminoglycoside nucleotidyltransferase AadA2* |
| *sul1* | NG_048082.1 | 100.0 | sulfonamide-resistant dihydropteroate synthase Sul1 |
| *sul2* | NG_051852.1 | 100.0 | sulfonamide-resistant dihydropteroate synthase Sul2 |
| *sul3* | NG_048120.1 | 100.0 | sulfonamide-resistant dihydropteroate synthase Sul3 |
| *oqxA* | NG_048024.1 | 100.0 | multidrug efflux RND transporter periplasmic adaptor subunit OqxA |
| *oqxB* | NG_048025.1 | 100.0 | multidrug efflux RND transporter permease subunit OqxB |
| *floR* | NG_047877.1 | 100.0 | chloramphenicol/florfenicol efflux MFS transporter FloR |
| *cmlA1* | NG_047648.1 | 100.0 | chloramphenicol efflux MFS transporter CmlA1 |
| *catB3* | NG_047604.1 | 100.0 | type B-3 chloramphenicol O-acetyltransferase CatB3 |
| *tetB* | NG_048163.1 | 100.0 | tetracycline efflux MFS transporter Tet(B) |
| *arr-3* | NG_048581.1 | 100.0 | NAD(+)--rifampin ADP-ribosyltransferase Arr-3 |
| *bla*_OXA-1_ | NG_049392.1 | 100.0 | oxacillin-hydrolyzing class D beta-lactamase OXA-1 |
| *bleO* | NG_047557.1 | 96.8 | bleomycin binding protein |

Table S5 Strain virulence information prediction

| Gene category | gene |  |
| --- | --- | --- |
| SPI-1 | *invH*、*invF*、*invG*、*invE*、*invA*、*invB*、*invC*、*invI*、*invJ*、*spaO*、*spaP*、*spaQ*、*spaR*、*spaS*、*sopD*、*sopE2*、*sopB*、*sopD2*、*sopA*、*sipB*、*sipC*、*sipD*、*sipA*、*sicA*、*sicP*、*sptP*、*prgH*、*prgI*、*prgJ*、*prgK*、*orgA*、*orgB*、*orgC*、*avrA*、*slrP*、*steB*、*steA* |  |
| SPI-2 | *gogB*、*sseL*、*sseI*、*pipB*、*ssaB*、*ssaC*、*ssaD*、*ssaE*、*sseA*、*sseB*、*sscA*、*sseC*、*sseD*、*sseE*、s*s*cB、*sseF*、*sseG*、*ssaG*、*ssaH*、*ssaI*、*ssaJ*、*ssaK*、*ssaL*、*ssaM*、*ssaV*、*ssaN*、*ssaO*、*ssaP*、*ssaQ*、*ssaR*、*ssaS*、*ssaT*、*ssaU*、*pipB2*、*sseK1*、*steC*、*sseJ*、*sifB*、*sseK2*、*sspH2*、*sifA* |  |
| SPI-3 | *mgtC*、*mgtB* | |
| Fimbriae | *csgG*、*csgF*、*csgE*、*csgD*、*csgB*、*csgA*、*csgC*、*fimF*、*fimH*、*fimD*、*fimC*、*fimI*、*lpfA*、*lpfB*、*lpfC*、*lpfD*、*lpfE* | |
| Others | *ompA*、*entB*、*entA*、*fepG*、*fepC*、*ratB*、*sinH*、*mig*-*14*、*sodCI*、*misL* | |

Table S6 Results of plasmid replicon prediction of strain

| Gene | Overlapping clone group | Starting site | Termination site |
| --- | --- | --- | --- |
| IncHI2A_1 | NODE_15 | 9 735 | 10 364 |
| pKPC-CAV1321_1 | NODE_49 | 4 079 | 4 673 |
| IncHI2_1 | NODE_49 | 5 292 | 5 618 |

Table S7 Prediction results of mobile elements

| Gene | Overlapping clone group | Starting site | Termination site |
| --- | --- | --- | --- |
| IS*Ec39* | NODE_16 | 5 342 | 6 641 |
| IS*Sen1* | NODE_39 | 20 683 | 21 994 |
| IS*Ec42* | NODE_41 | 9 749 | 11 038 |
| IS*Rle7* | NODE_42 | 2 524 | 3 414 |
| IS*Aba1* | NODE_42 | 5 806 | 6 985 |
| IS*Vsa3* | NODE_42 | 8 371 | 9 347 |
| IS*Vsa3* | NODE_42 | 12 655 | 13 499 |
| IS*1006* | NODE_42 | 13 500 | 14 318 |
| IS*10L* | NODE_44 | 4 812 | 6 140 |
| IS*2* | NODE_73 | 1 | 1 331 |
| IS*26* | NODE_80 | 1 | 820 |
| IS*1R* | NODE_81 | 1 | 768 |
| IS*200F* | NODE_83 | 3 | 711 |

Table S8 Colonization of *Salmonella* in Mice

| Grouping | Before attacking bacteria | | | After bacterial attack | | |
| --- | --- | --- | --- | --- | --- | --- |
|  | Number of samples  (copies) | *Salmonella* detection rate  (strains) | *Salmonella* detection rate  (%) | Number of samples  (copies) | *Salmonella* detection rate  (strains) | *Salmonella* detection rate  (%) |
| Group A | 118 | 0 | 0 | 175 | 174 | 99.4 |
| Group B | 118 | 0 | 0 | 175 | 175 | 100.0 |
| Group C | 118 | 0 | 0 | 175 | 175 | 100.0 |
| Group D | 96 | 0 | 0 | 123 | 0 | 0 |

Table S9 The weight gain of mice in the four groups before and after the experiment

| Group | Initial average weight (g) | End average weight (g) | Difference (g) |
| --- | --- | --- | --- |
| Group A | 22.2 | 26.8 | +4.7 |
| Group B | 23.5 | 29.0 | +5.5 |
| Group C | 22.5 | 26.5 | +3.9 |
| Group D | 23.1 | 32.4 | +9.3 |

Table S10 Alpha diversity indices of the small intestinal microbiota among experimental groups.

| Group | Community richness index | | | Community diversity index | | Community evenness index | **Community coverage index** |
| --- | --- | --- | --- | --- | --- | --- | --- |
|  | ACE | Chao1 | Sobs | Shannon | Simpson | Pielou_e | Coverage |
| Abx_Pre | 780.00 ± 179.27 | 790.08 ± 178.11 | 796.49 ± 176.32 | 4.84 ± 0.37 | 0.04 ± 0.01 | 0.73 ± 0.03 | 1.00 ± 0.00 |
| SE | 62.33 ± 30.35 | 88.44 ± 30.95 | 76.71 ± 30.03 | 0.93 ± 0.47 | 0.57 ± 0.24 | 0.23 ± 0.11 | 1.00 ± 0.00 |
| FMT | 140.00 ± 96.60 | 167.71 ± 113.56 | 164.47 ±112.93 | 1.98 ± 0.48 | 0.24 ± 0.07 | 0.41 ± 0.05 | 1.00 ± 0.00 |
| Abx_Tx | 78.67 ± 28.01 | 99.91 ± 35.58 | 91.81 ± 36.67 | 1.59 ± 0.17 | 0.29 ± 0.05 | 0.37 ± 0.01 | 1.00 ± 0.00 |
| Unt | 64.00 ± 21.00 | 85.62 ± 32.48 | 79.29 ± 26.16 | 1.13 ± 0.38 | 0.48 ± 0.19 | 0.27 ± 0.07 | 1.00 ± 0.00 |
| FMT_Rec | 107.67 ± 44.00 | 123.02 ± 48.96 | 119.57 ± 47.97 | 1.83 ± 0.33 | 0.25 ± 0.08 | 0.39 ± 0.04 | 1.00 ± 0.00 |
| Abx_Rec | 100.33 ± 37.98 | 132.92 ± 32.82 | 122.71 ± 32.46 | 1.95 ± 0.39 | 0.21 ± 0.07 | 0.42 ± 0.05 | 1.00 ± 0.00 |
| Unt_Rec | 71.33 ± 12.90 | 80.85 ± 17.75 | 77.68 ± 16.74 | 1.69 ± 0.16 | 0.27 ± 0.02 | 0.40 ± 0.02 | 1.00 ± 0.00 |
| Ctrl_D0 | 172.00 ± 67.56 | 229.94 ± 60.97 | 217.45 ± 69.38 | 1.72 ± 0.57 | 0.33 ± 0.18 | 0.33 ± 0.09 | 1.00 ± 0.00 |
| Ctrl_D1 | 251.33 ± 44.02 | 302.71 ± 53.19 | 286.62 ± 50.40 | 2.32 ± 0.35 | 0.19 ± 0.04 | 0.42 ± 0.05 | 1.00 ± 0.00 |
| Ctrl_D2 | 153.00 ± 45.51 | 189.84 ± 69.90 | 178.29 ± 68.91 | 1.67 ± 0.31 | 0.33 ± 0.15 | 0.34 ± 0.08 | 1.00 ± 0.00 |
| Ctrl_D3 | 112.00 ± 32.79 | 191.34 ± 50.16 | 161.07 ± 41.88 | 1.54 ± 0.52 | 0.35 ± 0.16 | 0.32 ± 0.10 | 1.00 ± 0.00 |
| Ctrl_D4 | 232.00 ± 47.15 | 285.91 ± 68.90 | 294.41 ± 86.64 | 2.24 ± 0.18 | 0.20 ± 0.02 | 0.41 ± 0.02ᵃ | 1.00 ± 0.00 |
| Kruskal-Wallis P value | 0.006 | 0.004 | 0.004 | 0.010 | 0.023 | 0.018 | 0.009 |
| Effect size, ε² | 0.602 | 0.663 | 0.639 | 0.548 | 0.447 | 0.475 | 0.555 |
| Magnitude | large | large | large | large | large | large | large |

Note. Values are presented as mean ± standard deviation (n = 3). Overall differences among the 13 experimental groups were assessed by Kruskal-Wallis tests, followed by Dunn's post hoc analysis with Benjamini-Hochberg correction for pairwise comparisons. Effect sizes were calculated as epsilon-squared (ε² = [H − k + 1]/[n − k]) and interpreted as negligible (< 0.01), small (0.01–0.059), moderate (0.06–0.139), or large (≥ 0.14).

Table S11 Standardized effect sizes for key pairwise comparisons of alpha diversity indices.

| Comparison | Shannon Cohen's *d* | 95% CI | Chao1 Cohen's *d* | 95% CI |
| --- | --- | --- | --- | --- |
| SE vs Ctrl_D1 | −3.40 | −5.90 to −0.90 | −5.06 | −8.34 to −1.78 |
| FMT_Rec vs Unt_Rec | 0.52 | −1.11 to 2.15 | 1.17 | −0.57 to 2.90 |
| FMT_Rec vs Ctrl_D4 | −1.54 | −3.37 to 0.28 | −2.50 | −4.63 to −0.36 |
| Unt_Rec vs Ctrl_D4 | −3.18 | −5.58 to −0.77 | −3.47 | −6.01 to −0.94 |

Note. Cohen's d was calculated as the mean difference between two groups divided by the pooled standard deviation: d = (M₁ − M₂) / SDₚₒₒₗₑₒ, where SDₚₒₒₗₑₒ = √[((n₁ − 1)SD₁² + (n₂ − 1)SD₂²) / (n₁ + n₂ − 2)]. The 95% confidence intervals (CIs) were estimated using the standard error approximation: SE = √[(n₁ + n₂)/(n₁n₂) + d²/(2(n₁ + n₂))]. Effect size magnitudes were interpreted according to conventional thresholds: |d| < 0.2 (negligible), 0.2–0.5 (small), 0.5–0.8 (medium), and ≥ 0.8 (large). Due to the small sample size per group (n = 3), the 95% CIs are wide; therefore, these estimates should be interpreted as indicative of biological magnitude rather than precise population parameters.

Table S12 Colon Intestinal Flora Alpha Diversity of Mice in Each Group

| Group | Community richness index | | | Community diversity index | | Community evenness index | **Community coverage index** |
| --- | --- | --- | --- | --- | --- | --- | --- |
|  | ACE | Chao1 | Sobs | Shannon | Simpson | Pielou_e | Coverage |
| Abx_Pre | 794.86 ± 275.06 | 796.87 ± 270.78 | 760.67 ± 253.26 | 3.23 ± 0.99 | 0.24 ± 0.18 | 0.49 ± 0.16 | 1.00 ± 0.00 |
| SE | 99.98 ± 3.00 | 97.54 ± 7.76 | 78.00 ± 10.00 | 1.85 ± 0.44 | 0.24 ± 0.08 | 0.42 ± 0.09 | 1.00 ± 0.00 |
| FMT | 147.26 ± 67.38 | 141.21 ± 61.16 | 124.67 ± 59.37 | 2.29 ± 0.33 | 0.19 ± 0.07 | 0.48 ± 0.02 | 1.00 ± 0.00 |
| Abx_Tx | 116.66 ± 48.53 | 105.23 ± 47.83 | 94.33 ± 43.10 | 2.45 ± 0.18 | 0.13 ± 0.00 | 0.55 ± 0.02 | 1.00 ± 0.00 |
| Unt | 70.54 ± 16.89 | 65.53 ± 18.25 | 61.00 ± 16.64 | 1.95 ± 0.35 | 0.23 ± 0.07 | 0.47 ± 0.06 | 1.00 ± 0.00 |
| FMT_Rec | 261.02 ± 67.93 | 262.55 ± 67.32 | 216.00 ± 53.84 | 2.92 ± 0.29 | 0.10 ± 0.03 | 0.55 ± 0.03 | 1.00 ± 0.00 |
| Abx_Rec | 157.56 ± 25.89 | 146.69 ± 29.65 | 127.33 ± 36.30 | 2.68 ± 0.29 | 0.12 ± 0.06 | 0.56 ± 0.03 | 1.00 ± 0.00 |
| Unt_Rec | 71.84 ± 9.58 | 77.81 ± 8.67 | 64.00 ± 12.29 | 2.03 ± 0.41 | 0.20 ± 0.07 | 0.49 ± 0.08 | 1.00 ± 0.00 |
| Ctrl_D0 | 529.45 ± 50.68 | 509.02 ± 32.61 | 402.33 ± 62.96 | 3.28 ± 0.48 | 0.09 ± 0.03 | 0.55 ± 0.07 | 1.00 ± 0.00 |
| Ctrl_D1 | 477.30 ± 114.69 | 450.30 ± 110.51 | 389.67 ± 104.71 | 3.08 ± 0.58 | 0.12 ± 0.06 | 0.52 ± 0.07 | 1.00 ± 0.00 |
| Ctrl_D2 | 445.58 ± 86.60 | 420.61 ± 104.52 | 343.33 ± 109.74 | 2.94 ± 0.63 | 0.15 ± 0.05 | 0.50 ± 0.08 | 1.00 ± 0.00 |
| Ctrl_D3 | 568.54 ± 71.00 | 552.66 ± 47.30 | 467.33 ± 41.55 | 3.57 ± 0.07 | 0.07 ± 0.01 | 0.58 ± 0.01 | 1.00 ± 0.00 |
| Ctrl_D4 | 508.80 ± 67.77 | 506.00 ± 74.51 | 430.33 ± 58.94 | 3.45 ± 0.35 | 0.08 ± 0.03 | 0.57 ± 0.04 | 1.00 ± 0.00 |
| Kruskal-Wallis P value | <0.001 | <0.001 | <0.001 | 0.010 | 0.040 | 0.220 | <0.001 |
| Effect size, ε² | 0.860 | 0.874 | 0.858 | 0.551 | 0.375 | 0.131 | 0.813 |
| Magnitude | large | large | large | large | large | moderate | large |

Note. Values are presented as mean ± standard deviation (n = 3). Overall differences among the 13 experimental groups were assessed by Kruskal-Wallis tests, followed by Dunn's post hoc analysis with Benjamini-Hochberg correction for pairwise comparisons. Effect sizes were calculated as epsilon-squared (ε² = [H − k + 1]/[n − k]) and interpreted as negligible (< 0.01), small (0.01–0.059), moderate (0.06–0.139), or large (≥ 0.14).

Table S13 Standardized effect sizes for key pairwise comparisons of alpha diversity indices.

| Comparison | Shannon Cohen's d | 95% CI | Chao1 Cohen's d | 95% CI |
| --- | --- | --- | --- | --- |
| SE vs Ctrl_D1 | −2.40 | −4.50 to −0.30 | −4.50 | −7.51 to −1.49 |
| Abx_Pre vs Ctrl_D1 | 0.19 | −1.42 to 1.79 | 1.68 | −0.18 to 3.54 |
| Unt vs Ctrl_D3 | −6.42 | −10.38 to −2.45 | −13.59 | −21.44 to −5.74 |
| FMT vs Ctrl_D3 | −5.35 | −8.77 to −1.93 | −7.53 | −12.07 to −2.98 |
| Abx_Tx vs Ctrl_D3 | −8.11 | −12.97 to −3.25 | −9.41 | −14.96 to −3.85 |
| Unt_Rec vs Ctrl_D4 | −3.72 | −6.36 to −1.07 | −8.07 | −12.91 to −3.23 |
| FMT_Rec vs Ctrl_D4 | −1.65 | −3.50 to 0.20 | −3.43 | −5.94 to −0.91 |
| Abx_Rec vs Ctrl_D4 | −2.40 | −4.49 to −0.30 | −6.34 | −10.26 to −2.41 |
| FMT_Rec vs Unt_Rec | 2.51 | 0.37 to 4.64 | 3.85 | 1.15 to 6.55 |

**Note. Cohen's d was calculated as the mean difference between two groups divided by the pooled standard deviation: d = (M₁ − M₂) / SDₚₒₒₗₑₒ, where SDₚₒₒₗₑₒ = √[((n₁ − 1)SD₁² + (n₂ − 1)SD₂²) / (n₁ + n₂ − 2)]. The 95% confidence intervals (CIs) were estimated using the standard error approximation: SE = √[(n₁ + n₂)/(n₁n₂) + d²/(2(n₁ + n₂))]. Effect size magnitudes were interpreted according to conventional thresholds: |d| < 0.2 (negligible), 0.2–0.5 (small), 0.5–0.8 (medium), and ≥ 0.8 (large). Due to the small sample size per group (n = 3), the 95% CIs are wide; therefore, these estimates should be interpreted as indicative of biological magnitude rather than precise population parameters.**.

**Fig. S1. Histopathological changes in intestinal tissues of mice under different treatments.** Representative H&E-stained sections of the duodenum, colon, and cecum are shown. The left panel shows intestinal tissues from antibiotic-treated mice (Abx), mice after antibiotic withdrawal/recovery (Abx_Rec), untreated infected mice (Unt), and untreated recovery mice (Unt_Rec). The right panel shows cecal tissues from control mice at day 0 (Ctrl_D0), mice infected with *Salmonella* enteritidis (SE), FMT-treated mice (FMT), and mice after recovery following FMT treatment (FMT_Rec). Images were captured at 400× magnification. Scale bars = 100 μm.
